# Revisiting the Role of Transcription Factors in Coordinating the Defense Response Against *Citrus Bark Cracking Viroid* Infection in Commercial Hop (*Humulus Lupulus* L.)

**DOI:** 10.3390/v11050419

**Published:** 2019-05-05

**Authors:** Vishnu Sukumari Nath, Ajay Kumar Mishra, Atul Kumar, Jaroslav Matoušek, Jernej Jakše

**Affiliations:** 1 Department of Molecular Genetics, Institute of Plant Molecular Biology, Biology Centre of the Czech Academy of Sciences, Branišovská 31, 37005 České Budějovice, Czech Republic; sukumari.nath@umbr.cas.cz (V.S.N.); ajaymishra24@umbr.cas.cz (A.K.M.); atul.kumar@umbr.cas.cz (A.K.); jmat@umbr.cas.cz (J.M.); 2 Department of Agronomy, Biotechnical Faculty, University of Ljubljana, Jamnikarjeva 101, SI-1000 Ljubljana, Slovenia

**Keywords:** transcriptional reprogramming, viroid pathogenesis, hop stunt disease, management

## Abstract

Transcription factors (TFs) play a major role in controlling gene expression by intricately regulating diverse biological processes such as growth and development, the response to external stimuli and the activation of defense responses. The systematic identification and classification of TF genes are essential to gain insight into their evolutionary history, biological roles, and regulatory networks. In this study, we performed a global mining and characterization of hop TFs and their involvement in *Citrus bark cracking viroid* CBCVd infection by employing a digital gene expression analysis. Our systematic analysis resulted in the identification of a total of 3,818 putative hop TFs that were classified into 99 families based on their conserved domains. A phylogenetic analysis classified the hop TFs into several subgroups based on a phylogenetic comparison with reference TF proteins from *Arabidopsis thaliana* providing glimpses of their evolutionary history. Members of the same subfamily and subgroup shared conserved motif compositions. The putative functions of the CBCVd-responsive hop TFs were predicted using their orthologous counterparts in *A. thaliana*. The analysis of the expression profiling of the CBCVd-responsive hop TFs revealed a massive differential modulation, and the expression of the selected TFs was validated using qRT-PCR. Together, the comprehensive integrated analysis in this study provides better insights into the TF regulatory networks associated with CBCVd infections in the hop, and also offers candidate TF genes for improving the resistance in hop against viroids.

## 1. Introduction

Plants confront numerous biotic stresses in their natural habitat through a compendium of well-tuned surveillance systems including a sophisticated genetic defense mechanism. The successful defense response in plants relies on the timely and accurate detection of the invading pathogen and the subsequent induction of the components of the defense response pathways to ward off the pathogens. The transcriptional reprogramming is one of the key events in a plant’s defense response and involves a myriad of complex molecular, biochemical, and physiological changes, occurring in a highly synchronized manner largely governed by transcription factors (TFs) [1]. The lineage-specific TFs constitute the repertoire of master regulators, which mediate the transcriptional regulations by binding a specific DNA sequence in response to an environmental stimulus and developmental cues in plants [1]. TFs are central to the modulation of gene expression and play distinctive roles in diverse biological processes such as developmental control, and the activation of signaling cascades in defense and stress responses, by intricately regulating the transcription of downstream targets with the interaction of co-regulators known as transcriptional regulators (TRs) [2]. Mounting studies suggested that several families of TFs play an important role in the regulation of the expression of the plant defense transcriptome [2,3]. For example, WRKY TFs, regarded as the core of the plant immune system, play an indispensable roles in pathogen defense and phytohormone signaling [4]. Members of MYB/bHLH TFs or their complexes regulate distinct cellular processes such as responses to biotic stress, hormone signaling and cell death [5,6]. Similarly, bZIP TFs play critical roles in numerous cellular functions, including the regulation of stress-responsive pathways and the pathogen defense [7]. A recent explosion in the field of genomics and transcriptomics has enabled the family-wide identification and characterization of a large repertoire of TFs involved in a diverse array of biological processes in plants, such as the MYB, ADP-ribosylation factor, WRKY [8,9,10]; NAC [11]; bHLH [12]; Dof [13] and bZIP [14]. These studies have opened new avenues for exploring and exploiting TFs, to improve disease resistance in plants against pathogens.

Viroids are the smallest (246–401 nt) autonomous plant pathogens, with the lowest biological complexity currently known. Their genome is approximately tenfold smaller than viruses and consists of single-stranded, circular, highly structured RNA that lacks a protein-coding capacity [15]. They are classified into two taxonomic families based on their biological, structural, and biochemical properties; the *Pospiviroidae* and the *Avsunviroidae*, whose members replicate in the nucleus and chloroplast respectively [16]. Viroids are cosmopolitan in their distribution and are etiological agents of diseases affecting a broad range of plants, including herbaceous, agronomic, ornamental and tree crops. In hosts, they cause minor to severe symptoms leading to plant death, depending on the host genome complexity and the aggressiveness of the viroid species [15]. In addition to symptomatic infections, viroids also cause latent infections without any noticeable symptoms. The simplicity of the viroid genome and their high rates of per-base in vivo mutation compared to other nucleic acid-based pathogens makes them an intriguing model for studying the RNA structure-functional relationship [15].

Hop (*Humulus lupulus*), a member of the *Cannabaceae* family, is a dioecious, climbing, herbaceous, perennial plant that is native to Europe, Asia, and North America [17]. Hop plants are commercially grown for their cones (strobiles) that are rich sources of secondary metabolites such as essential oils, bitter acids, and prenylated flavonoids. These compounds serve as essential raw material for the beer production industry, providing distinctive bitterness, flavor and aroma. In addition, the hop plant possesses several medicinal and soporific effects and are used as edible delicacies. Diseases caused by viroids impose significant global challenges for the commercial production of hop [18]. This includes *Hop stunt viroid* (HSVd) *Apple fruit crinkle viroid* (AFCVd), *Hop latent viroid* (HLVd), and *Citrus bark cracking viroid* (CBCVd) [19,20], of which the CBCVd infection is the most aggressive. Though several non-hosts are identified for viroids, no durable natural resistant sources have been discovered in hop species. In hop, macroscopic symptoms of a viroid infection include leaf distortion and mottling, stunting, chlorotic or necrotic spots, epinasty, vein discoloration and clearing, malformation of flowers and cones, scaling and cracking of the bark, and death of the plant in the case of CBCVd infections. The current management practices for viroid disease include detection and eradication, as well as agronomic maneuvers.

Viroid pathogenesis is extremely complex and is influenced by both the host and viroid genomes, resulting in mild or asymptomatic to severe symptoms [17]. Our recent transcriptome analysis of the CBCVd-hop interaction has revealed a massive transcriptional reprogramming of the genes involved in the immune response, sugar metabolism, photosynthesis, and phytohormone signaling pathways [21]. Nevertheless, transcriptional reprogramming is governed by TFs, and in this context it is crucial to delineate the pivotal role played by TFs in finely coordinating diverse regulatory networks. The involvement of hop TFs in the viroid pathogenesis is still enigmatic. In this study, we systematically identified hop TFs, categorized them in various TF families according to their conserved domains and examined their phylogenetic relationships to those of the model plant *A. thaliana*. Furthermore, we analyzed the expression signatures of a subset of hop TF families during CBCVd infection using the digital gene expression and a qRT-PCR assay. To the best of our knowledge, this study represents pioneering attempt to comprehensively identify and classify CBCVd-responsive hop TFs and provides valuable information about their involvement in hop-viroid pathogenesis.

## 2. Materials and Methods

### 2.1. Identification of Hop Transcription Factors

The previously published hop genome and CBCVd-responsive transcriptome profile generated at 412 days post-inoculation (dpi) [21,22] were used for the global mining of hop TFs. The comprehensive hop TFs database was constructed based on a homology search of the available transcriptome datasets of hop against the plant TF database (https://plantgrn.noble.org/PlantTFcat/) [23], which resulted in the classification of hop transcripts into various TF families. To validate this classification, the Hidden Markov Model (HMM) profile for each of the identified TF families was downloaded from the Pfam protein family database (http://pfam.sanger.ac.uk/), and a HMMER search (http://hmmer.janelia.org/) was performed to identify the presence of conserved family domains in identified TF sequences. To filter out false positives, we cross-checked all TF sequences using a batch CD-search in the NCBI conserved domain database (https://www.ncbi.nlm.nih.gov/Structure/cdd/cdd.shtml) and SMART database (smart.embl-heidelberg.de). All non-redundant sequences encoding complete TF domains were considered to be putative TF genes. Subsequently, a nucleotide-protein (blastx) (https://blast.ncbi.nlm.nih.gov/Blast.cgi) search was performed using the nucleotide sequences of the identified hop TFs to examine their homology with related sequences in the NCBI protein database. 

### 2.2. Protein Characterization, Conserved Motifs and Gene Ontology Identification

The ProtParam tool available on the Expert Protein Analysis System (ExPASy) proteomics server (http://web.expasy.org/protparam/) was used to compute the isoelectric points (pI) and molecular weight of the hop TFs. The publicly available web server CELLO v 2.5 (http://cello.life.nctu.edu.tw/) [24] was employed for the prediction of the localization of hop TFs.

The Multiple Expectation-maximization for Motif Elicitation (MEME) programme (http://meme-suite.org/tools/meme) was used to statistically identify the additional conserved motifs of the hop TFs with the following parameters: maximum number of motifs: 10, and motif length set to 6–100 and motif sites to 2–120; The distribution of one single motif was “any number of repetitions”, and the other search parameter was “search given strand only”.

To gain insights into the molecular function, biological process and cellular component of the identified hop TFs, a Gene ontology (GO) annotation was performed using the Blast2GO command line tool v 4.1 [25]. The TF nucleotide sequences were BLASTX searched against the non-redundant protein database of plants in NCBI under the default parameters. The GO terms associated to hit sequences were obtained by mapping and InterPro annotation. 

### 2.3. Phylogenetic Affinity of Hop TFs

To deduce the phylogenetic relationships of hop TFs, the amino acid sequences were aligned along with the reference TF protein sequences of *A. thaliana* downloaded from the plant transcription factor database (http://planttfdb.cbi.pku.edu.cn/) using the MUSCLE program [26] with default parameters. Based on the alignment, unrooted phylogenetic trees were constructed for each of the TF families using MEGA v 7.0 software [27]. The criterion used for the tree construction were the Neighbor-Joining method, the JTT model, and complete deletion. The confidence level of the monophyletic groups was assessed using a bootstrap analysis based on 1000 re-samplings. The tree was edited with the Figtree tool (http://tree.bio.ed.ac.uk/software/figtree/).

### 2.4. The CBCVd-Responsive Expression Datasets Availability and Analyses of Differentially Regulated TFs 

To gain an insight into the digital expression profiles of hop TFs during CBCVd infection, we have used our recently published CBCVd-responsive transcriptome [21]. Briefly, we mapped the clean reads to the unigenes dataset and normalized to the number of fragments per kilobase of exon per million mapped fragments (FPKM) by expectation maximization (RSEM) protocol using the Trinity software package [28]. The obtained count value was exported to the DESeq2 R package [29] to determine differentially expressed gene transcripts (DEG) using the Benjamini and Hochberg approach [30] for controlling the false discovery rate (FDR). The expression of TF encoding unigenes with an FDR adjusted *p*-value ≤ 0.05 and at least a two-fold change (≥2 or ≤2) was considered to be significantly differentially expressed between the CBCVd-infected and control libraries. The TF gene expression was visualized using a heatmap with clustering drawn using the online Clustvis tool [31].

### 2.5. Identification of Orthologous Genes and Protein Interactions

The identification of orthologous genes in well-characterized model plants helps in predicting the putative functions of newly identified genes in non-model plants such as hop. Therefore, the *A. thaliana* genes that are closest (orthologous) to the hop TFs were determined using an eggnog-mapper [32] based on the eggNOG 4.5 orthology data [33]. 

TFs often interact among one another during transcriptional reprogramming. To dissect the plausible interactions of hop TF proteins, a protein-protein interaction (PPI) analysis was performed using the STRING v 11 (http://string-db.org) [34] database in the COG (Clusters of Orthologous Group) mode, using the best-assigned COGs of *A. thaliana*. The interactions with a confidence score of ≥ 0.7 and based on co-expression and experiment conditions were used to construct the PPI network. The top 10 interactions were identified using the cytoHubba package [35], and the interaction network was visualized using the Cytoscape v.3.7.0 [36]. 

### 2.6. RNA Extraction and qRT-PCR Validation of Selected Hop TFs

The total RNA from the hop CBCVd-infected and control leaf samples was isolated by Concert™ Plant RNA Reagent (Invitrogen, Carlsbad, CA, USA) following the manufacturer’s protocol. The RNA concentration and quality were assessed using a Nanodrop ND-1000 spectrophotometer (NanoDrop Technologies Inc., Waltham, MA, USA). The total RNA was treated with the TURBO DNA-*free*™ Kit (Invitrogen, Carlsbad, CA, USA) to remove genomic DNA traces, and first strand cDNA was synthesized using the Superscript III Kit (Invitrogen, Carlsbad, CA, USA) from 1 μg of total RNA, according to the manufacturer’s protocol. The obtained cDNA was diluted 10-fold and used as the template for the qRT-PCR analysis.

Eleven pairs of specific primers were used to study the expression of the selected hop TFs in the CBCVd infected leaf tissues of the hop plants. These TFs were carefully selected based on their reported defense response roles in various stages of the plant-pathogen interaction (Table S1). TF gene-specific primers were designed using the PerlPrimer software [37], and the specificity of the primer pairs was assessed in silico by performing an NCBI primer blast [38]. The qRT-PCR was performed in a CFX Connect™ Real-Time PCR Detection System (Bio-Rad, Hercules, CA, USA) using the TopBio SYBR master mix (TopBio, Prague, Czech Republic). The reactions were performed in 15 μL comprising of 7.5 μL of SYBR Green, 0.75 μL (10 μM) of forward and reverse primers, 3 μL of RNase free-water and 3 μL of cDNA. The PCR cycling regime was at 95 °C for 3 min, followed by 40 cycles of 95 °C for 15 s, 58 °C for 30 s and 72 °C for 30 s. The specificity of each primer pair was assessed using a melt curve at the end of the reaction. The threshold cycles (Ct) of each TF target were averaged for triplicate reactions, and the relative transcriptional changes in the gene expression levels (fold-change) were calculated through the comparative Ct (2^-ΔΔCt^) method [39] using DRH1 (DEAD-box ATPase-RNA-helicase) [40] as the reference gene. Three biological replicates were used for each sample.

## 3. Results

### 3.1. Identification and Classification of Hop TFs

With the aim of defining the hop TF gene families, we performed a global examination of the hop transcriptome using the plant TF database (https://plantgrn.noble.org/PlantTFcat/), which identified 3,818 non-redundant TFs that were classified into 99 TF families based on their conserved domains (Table 1). The blast analysis showed that most hop TFs shared the highest homology to *Morus notabilis*, with the similarity ranging from 69% to 99% (Table S2). A large proportion of the TFs identified in this study belonged to the well-characterized TF families (e.g., WRKY, AP2, bZIP, MYB, bHLH, and NAC). Since it is impossible to describe the exhaustive list of TFs and their plausible roles in a single communication, we have in this study adopted the selective method to characterize the role of the major TF families, viz. WRKY, AP2, bZIP, MYB, bHLH, and NAC, that play indispensable roles in the plant immune response [3,41,42].

### 3.2. Protein Characterization, Conserved Motifs and Gene Ontology Identification

The ExPASy analysis of the CBCVd-responsive hop TFs showed a range of variation in their molecular weight (7.2–96.3 kDa) and isoelectric point (4.5–9.89) (Table S2). The subcellular localization analysis of the hop TF proteins was predicted, and most of them were found to be localized in the nucleus (445, 95.91%), followed by the chloroplasts (11, 2.37%), cytoplasm (5, 1.08%) and mitochondria (3, 0.65%) (Table S3). 

The conserved and novel additional motifs in the hop TF protein sequence were identified using the online MEME analysis tool. The analysis revealed that all of the TFs possessed their characteristic conserved core family domains. Most TF members that were clustered in the same subfamilies displayed similar motif characteristic, suggesting that the protein architecture is conserved within a subfamily and might reflect functional similarities among the members (Figure S1). However, there were also some differences observed in the members among the subfamilies. Few subgroups had common motifs for all of the members, whereas other subgroups (e.g., AP2, WRKY TFs) possessed special motifs which could be attributed to their functional diversity (Figure S1).

**Figure 1:**
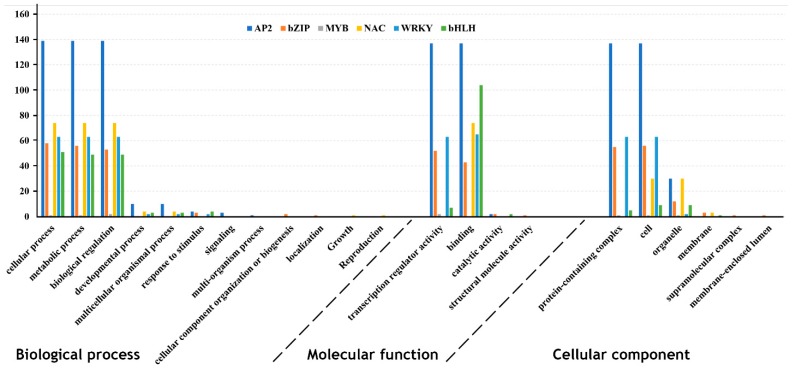
Gene ontology classification of the CBCVd-responsive hop TFs describing the molecular functions, biological processes and cellular components. The analysis was performed using the Blast2Go software (command line version).

The functional annotation of the 466 hop TFs revealed their association with a range of biological processes, molecular functions, and cellular component categories (Figure 1). In the biological process category, a predominant portion of the TFs was found to be involved in the cellular process, metabolic process, and in biological regulation (386) (Figure 1). The most highly enriched molecular function categories were related to binding (423), transcriptional regulator activity (261), and catalytic activity (6) (Figure 1). The cellular component category included terms related to the cell (296), organelle (84) and membrane (7).

### 3.3. Phylogenetic Affinity of Hop TFs

**Figure 2:**
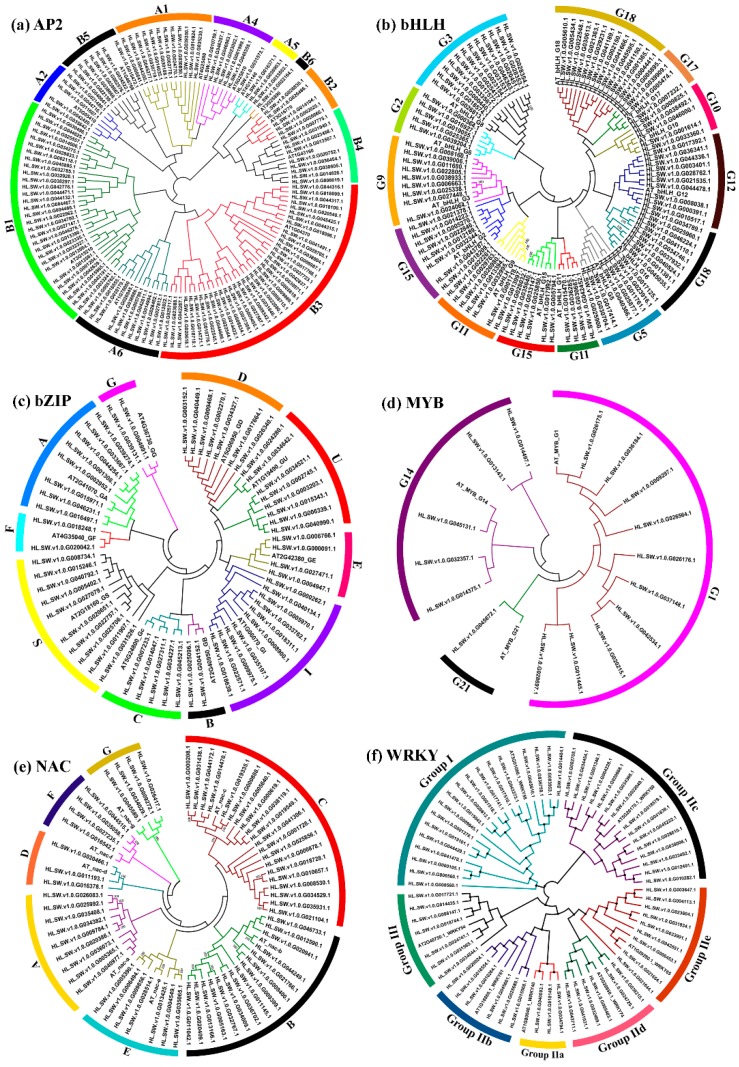
Unrooted phylogenetic tree representing the relationships and subfamily designations of the hop TF families along with the model species *A. thaliana*. The deduced amino acid sequences were aligned using the MUSCLE program, and the phylogenetic tree was constructed using the neighbor-joining method implemented in MEGA 7 software. The colored branch indicates the different subfamilies: (**a**) AP2, (**b**) bHLH, (**c**) bZIP, (**d**) MYB, (**e**) NAC and (**f**) WRKY.

To explore the phylogenetic relationships between the hop TFs and to clarify the evolutionary relations within the family, an unrooted phylogenetic tree was constructed, along with *A. thaliana* reference TF proteins. As is evident from Figure 2, the phylogram distributed the hop TFs into different clades based on their evolutionary relationships. Sakuma et al. (2002) [43] have described a total of 12 groups for the AP2/ERF family in *A. thaliana*. In our study, the 140 hop AP2/ERFs were grouped into 11 groups (A1, A2, A4, A5, A6, B1, B2, B3, B4, B5, and B6) with Group B3 forming the major clade with 37 members (Figure 2a). In *A. thaliana*, WRKY TFs have been classified into 3 main groups (Group I–III) (Eulgem et al. 2005) [44]. Here, the hop WRKY TFs were distributed into three major groups (Group I, Group IIa, Group IIb, Group IIc, Group IId, Group IIe, and Group III; Figure 2f), where Group I formed the major clade with 18 members. In the case of the *A. thaliana* bZIP family, 11 groups have been previously reported (Jakoby et al. 2002) [7]. Our phylogenetic tree categorized the bZIP TF proteins into 10 major groups (Figure 2c) with groups S and I forming the major clade with 18 members each. Among the 22 bHLH groups described in *A. thaliana* (Toledo-Ortiz et al. 2003) [6], the 106 hop bHLH TFs could be categorized into 13 groups (Figure 2b), which were further sub-grouped into smaller clades based on their phylogenetic affinity. Group 3 formed the major group with 12 members, followed by Group 12 with 10 members. Based on the previous classification, NAC TFs of *A. thaliana* were divided into 21 subfamilies (Zhu et al. 2012) [45]. In the present study, the hop NAC TFs were extensively distributed into 7 groups (viz. A, B, C, D, E, F, and G) (Figure 2e). The *A. thaliana* MYBs were extensively studied and classified into 22 groups by Stracke et al. (2001) [5]. The MYB phylogram clustered the 16 hop TFs into 3 groups (Figure 2d), with Group I forming the major clade.

### 3.4. Differential Modulation of Hop TFs

**Figure 3:**
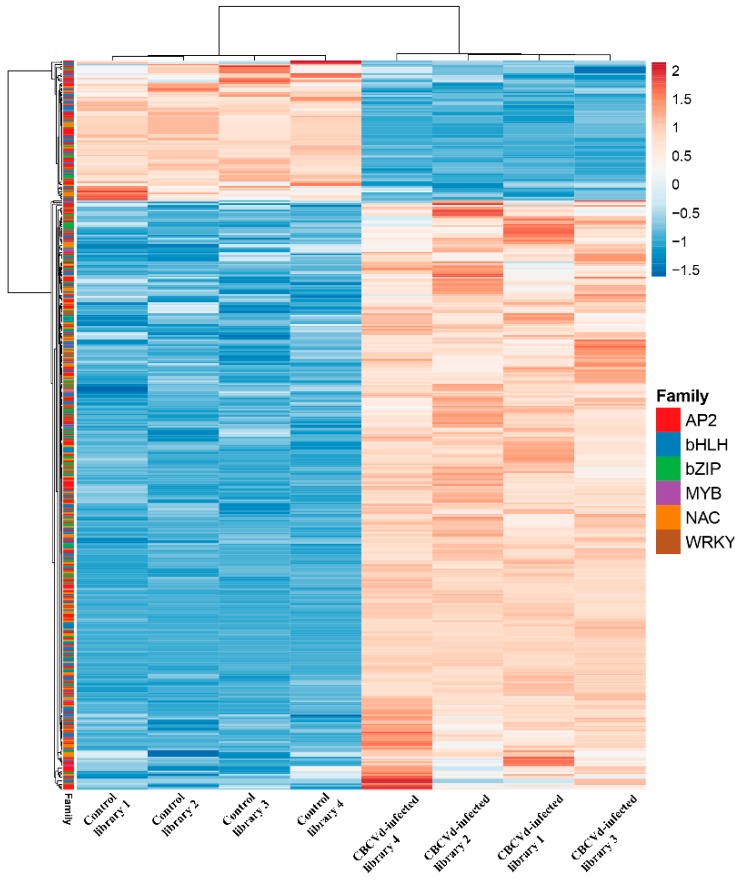
RNA-seq transcript abundance of *Citrus bark cracking viroid* (CBCVd) infected and control (healthy) hop plants. The values are expressed as the log2 value of average fragments per kilobase of exon per million fragments mapped (FPKM) per sample. The heatmap with clustering was drawn using the online Clustvis tool.

**Figure 4:**
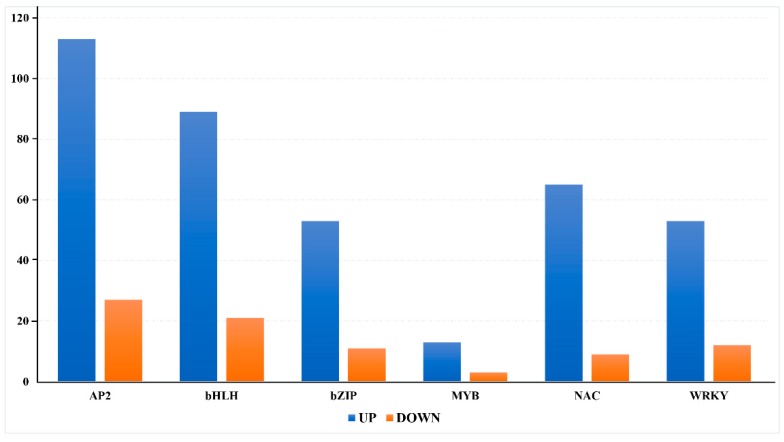
Summary statistics of the TF genes differentially modulated during CBCVd infection in the hop. TF genes with a fold change (≥+2 and ≤−2; *p*-value < 0.05) were considered to be significantly differentially modulated.

To understand the intricate differential modulation of hop TFs during CBCVd infection, we analyzed the TF digital gene expression levels in CBCVd-infected and control samples using FPKM. The result revealed the massive differential modulation of hop TFs, with FPKM values ranging from 0.14 to 894.55 in CBCVd infected libraries (Table S4). We constructed an expression profile heat map based on the expression data in different libraries of hop TFs (Figure 3). Most of the TFs (440, or 95%) depicted a significant differential expression pattern (*p* ≤ 0.05, logFC ≥ 2 or ≤ −2). Of the total 466 TFs, a total of 113 (AP2; 80.70%), 53 (bZIP; 82.8%), 85 (bHLH; 80.10%), 13 (MYB; 81.67%), 65 (NAC; 87.83%) and 53 (WRKY; 80.30%) TFs were upregulated, while 27, 11, 21, 3, 9 and 13 TFs were downregulated, respectively (Figure 4), in this study reflecting their concerted activator or repressor roles during CBCVd infection.

### 3.5. Identification of Orthologous Genes and Protein Interactions

Information regarding orthologous genes in well-characterized model species can help in predicting the putative functions of newly identified genes. Here, we surveyed orthologous genes from the well-characterized model plant *A. thaliana*, which helped us in predicting the putative functions of the hop TFs (Table S5).

**Figure 5:**
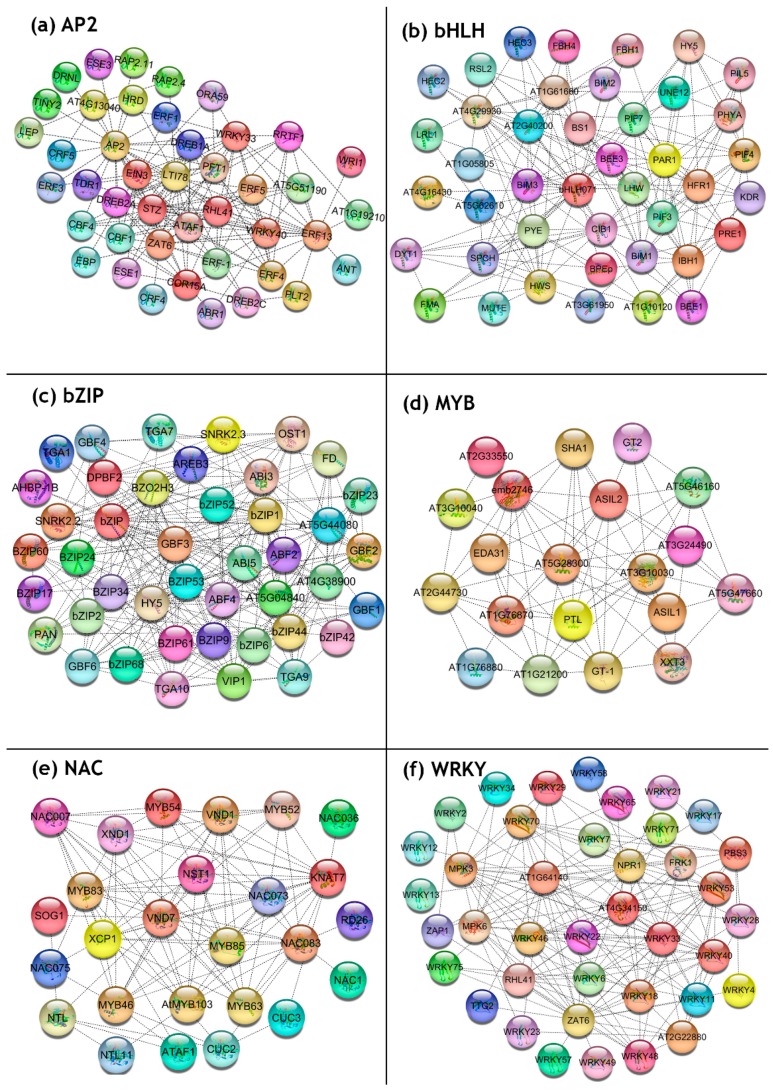
Network analysis of CBCVd-responsive hop TFs in a cluster of orthologous (COG) mode using the STRING v11.0 database showing the interconnected network of hop TFs with other proteins involved in the defense response, with a confidence score ≥0.7. The top ten interactions were extracted using the cytohubba app and visualized using Cytoscape software (**a**) AP2 family, (**b**) bHLH family, (**c**) bZIP family, (**d**) MYB, (**e**) NAC, and (**f**) WRKY.

TF proteins are known to interact with each other and regulate the transcription of downstream targets. Therefore, to predict the PPI among the CBCVd-responsive hop TFs, a protein-protein interaction analysis was performed using the STRING database, using *A. thaliana* as a model system. The analysis revealed that most of the CBCVd-responsive hop TF proteins were involved in multiple interactions, with more than one CBCVd-responsive TF protein belonging to the same or different TF family (Figure 5). For instance, NAC TFs were found to be actively interacting with MYB TFs; while AP2 TFs were largely involved in the interaction with WRKY and NAC TFs, suggesting a highly coordinated and programmed interaction of CBCVd-responsive hop TFs. Additionally, several TFs were predicted to be acting as hub genes (e.g., NAC83, MYB85, etc.) in the interaction network, reinforcing their crucial role in transcriptional reprogramming (Figure 5e,d) during CBCVd-infection in the hop. A detailed description of the functions of proteins represented in the interaction network is presented in Table S6.

### 3.6. qRT-PCR Validation of Selected Hop TFs

**Figure 6:**
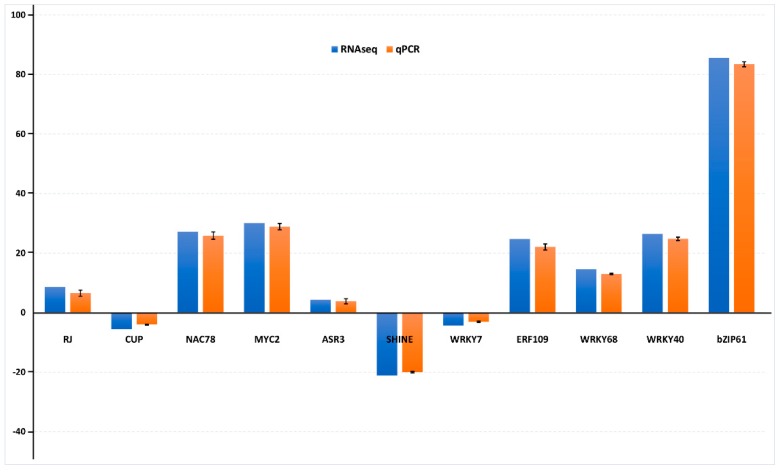
The quantitative real-time (qRT-PCR) expression profiles of 11 selected hop TF genes in response to CBCVd infection. The data were normalized to the (DEAD-box ATPase-RNA-helicase) DRH1 gene, and the vertical bars indicate the standard deviation.

Based on the orthologous relationships and putative known functions, we selected 11 different CBCVd-responsive hop TFs for the qRT-PCR validation. The analysis revealed a high degree of correlation between the RNA-Seq digital gene expression and the qPCR expression statistics (Figure 6), implying the high quality and reliability of our transcriptome datasets. In addition, the differential expression profile of the selected hop TFs further strengthens their candidature in transcriptional reprogramming during CBCVd infection in the hop. For instance, the WRKY (WRKY68&40), bZIP (bZIP61) and NAC (NAC78) TFs depicted a significant up-regulation, which was consistent in both the RNA-Seq and qRT-PCR analysis. Similarly, the CUP-SHAPED COTYLEDON 3 (NAC), SHINE 2 (AP2) and WRKY7 TFs showed a similar trend of down-regulation in the transcriptome and qRT-PCR analysis. 

## 4. Discussion

The rapid, large-scale and coordinated intracellular and tissue-wide reprogramming of gene expression at the transcriptional level constitutes a crucial step in mounting the immune response against invading pathogens in plants [46]. The fine-tuned, complex regulatory system consisting of TFs and co-regulatory proteins functions as important components in the tight regulation of the transcriptional reprogramming of genes associated with the plant defense response. The growing body of research over the past years has identified and characterized the intriguing roles of several TF gene families involved in the plant defense response. However, a larger proportion of these studies have been centered on model plant species such as *A. thaliana* [6,47], rice [48] and maize [49], while detailed systematic information on major TF families in a genomic context and their response against viroid pathogenesis is still enigmatic in the hop. In this study, the CBCVd-responsive transcriptome data generated in our laboratory, combined with in silico approaches, have enabled us to conduct a systematic characterization of TFs and their regulatory roles in defense responses against CBCVd infection in the hop. The combined approach enabled the prediction of 3818 TFs, along with their sequence features and the putative phylogenies of the largest families in the hop, which is significantly higher than the number of TFs (ca. 2000) identified in *A. thaliana* [47], suggesting that the high rate and their propensity for parallel expansion patterns is a consequence of an adaptive response against selection pressure [50]. The comparison of the TF gene families of hop with other plant species has shown considerable variations in the count, which could be attributed to genome complexity, the succession of genomic rearrangements and the shaping of the gene regulatory network during the evolution of organism complexity [51].

Moreover, the phylogenetic comparative method analysis was employed to further interrogate the evolutionary correlations and classification of TF proteins, which resulted in the clustering of the identified TF family of the hop into several subgroups according to their phylogenetic affinity toward all well-classified TF subgroups in *A. thaliana*, which suggested that the in silico approaches of the mining of TFs from the hop transcriptome dataset is comprehensive and reliable. In general, gene duplication and differentiation play a major role in the origin of new genes and gene functions [52]. Importantly, the DNA binding domains of TFs are extremely crucial for their biological functions. Accordingly, the classification of the TF gene family based on the phylogenetic trees may reflect the role of domains in the gene. Though the roles of most hop TFs remain to be elucidated, it is likely that members of a given group/subgroup share common evolutionary origins and might have similar physiological functions. Intriguingly, we observed that few hop TFs (e.g., AP2 and bZIP family TFs) that belonged to the same subfamily were clustered in different clades in the phylogenetic tree (Figure 2a,b), which could be attributed to the occurrence of duplication and divergent events in hop TF genes. The previous observation of the clustering of some MYB TFs of Foxtail millet in different subclades [53] corroborated our analysis.

It is generally accepted that overrepresented amino acid motifs in TFs tend to represent functional regions that are evolutionarily conserved across or within specific lineages [54] with similar biological functions. In this context, we analyzed additional conserved motifs outside the family domain in the hop TFs using the online MEME program. The analysis revealed that the majority of the TFs were grouped into the same subfamilies and shared a similar motif composition (Figure S1). The presence of highly conserved motifs among proteins of the same subfamily supports the notion that they are likely produced via gene expansion during plant evolution and that they are essential for the group-specific functions within subfamilies. However, a high divergence in the motif composition was also observed for TFs belonging to different groups, reflecting the complex protein architecture and diverse functions of hop TF proteins. Novel TFs are likely to emerge through the rearrangement into novel signature domains or through new combinations of existing domains [47]. Our results were in agreement with the previous reports of the identification of a considerable domain conservation within a TF subfamily but a divergence among the subfamilies [55,56,57]. In addition, the occurrence of some special motifs in a few subfamilies confirms the fact that domain-shuffling processes might have also have played a relatively significant role in the evolution of hop TFs. However, further studies are needed to confirm these observations.

Orthologues are defined as genes in different genomes that have been created by the splitting of taxonomic lineages and are likely to retain the same function [58,59]. It has been widely accepted that the functions of the newly identified genes could be putatively predicted according to their orthologous counterparts [60]. In this study, we predicted the functions of the identified hop TFs using the orthologous genes from the well-characterized model plant *A. thaliana*. A large proportion of the hop TFs was assigned to have putative functions in diverse biological processes such as hormonal signaling, developmental processes, and abiotic and biotic stress responses (Table S4).

In a given cell or organism, biological and physiological processes, including cell-to-cell interactions and metabolic and developmental control, are regulated by protein-protein interactions (PPI) between different TFs [61]. In particular, the expression of a diverse set of eukaryotic genes are regulated by relatively small numbers of TFs by their different combinations and under different stress conditions; the physical interactions among different combinations of TFs dictate and regulate the expression of specific genes [62]. Moreover, the analysis of interactions between transcription factors and other proteins also help in elucidating their role in different signaling cascades [63]. Several explicit evidences supported that the PPI network could be exploited for the manipulation of plants for disease resistance against pathogens [64,65]. Therefore, in this study, we have extensively investigated the putative protein-protein interactions (PPI) of CBCVd-responsive TFs to gain an understanding of the regulatory networks that modulate CBCVd-defense responses in the hop. In this study, the interaction network explicitly projected the complex interaction web of CBCVd-responsive hop TFs with diverse proteins involved in defense response, hormone signaling, and transcription. TFs act as sites of signal convergence in concert with other context-specific TFs and transcriptional co-regulators to determine the activation or repression of defense response pathways which ultimately establish sensory transcription regulatory networks that are required for plant immunity [3]. The PPI network explicitly revealed the interfamily interactions of hop CBCVd-responsive TFs (e.g., NAC TFs interacting with MYB and bHLH TFs (Figure 5e); AP2 TFs interacting with WRKY TFs (Figure 5a)), reinforcing a complex, interconnected and finely-tuned transcriptional reprogramming of hop TFs in activating downstream defense pathways against CBCVd infection. A growing body of evidence suggests that WRKY TFs respond to biotic stresses through the auto-regulation and cross-regulation of other WRKY TFs, while interacting with a diverse array of defense responsive genes, forming an interaction hub [61,66]. The presence of a highly interconnected hub of several WRKY TFs, along with their dominant interactions with the mitogen-activated protein kinase 3 (MAPK3) (a component of the kinase module that plays a pivotal role in the transduction of diverse extracellular stimuli, including biotic stress, in plants) and the Non-Expresser of Pathogenesis Related Gene 1 (NPR1), a receptor for the plant defense hormone salicylic acid (SA) (Figure 5f), provide strong evidence for the auto/cross-regulation phenomenon of WRKY TFs in our PPI network. In addition, the explicit interaction of WRKY TFs with NPR1 suggests the crucial role of a SA mediated defense response in CBCVd-infected hop plants. The involvement of Brassinosteroid pathway (BR) genes in the viroid interaction has been previously reported in PSTVd-infected tomato [67] and potato [68]. In agreement with the above reports, we observed the involvement of BR pathway genes in CBCVd infection of the hop, as evidenced by the profound interaction hub of BIM1 & 2 with the hop bHLH TFs (Figure 5b). Together, the PPI interactions presented here are crucial to an understanding of the transcriptional regulatory networks that operate in triggering defense responses in hop against CBCVd infection, and they provide glimpses of putatively activated defense pathways and candidate proteins interacting with them.

The successful activation of the plant defense signaling involves the coordinated regulation of multiple transcriptional regulators, as well as DNA-binding transcription factors and their regulatory proteins, that rapidly reprogram the transcription in the plant cell in response to biotic stress [46]. To gain a better understanding of the intricate differential modulation of CBCVd-responsive hop TFs, we conducted a digital gene expression profiling using the FPKM derived from the hop-CBCVd transcriptome dataset. Our results illustrated the massive transcriptional reprogramming of hop TFs during CBCVd infection, with a higher percentage of TFs depicting a significant up-regulation (Figure 4). It is worth noting that the AP2, bZIP, and WRKY families were found to be predominantly participating in the transcriptional reprogramming in response to CBCVd infection in the hop, as evidenced by their pronounced differential modulation (Table S3). The abundance of AP2, bZIP and WRKY TF families in the transcriptional reprogramming during viroid interaction has also been recently reported in tomato plants infected with *Potato spindle tuber viroid* (PSTVd) [69]. The AP2, bZIP, and WRKY TFs represent the largest repertoire of TF families, and considerable evidence suggested that they play pivotal roles in the regulatory networks controlling plant development, hormone signal transduction and disease resistance, in response to several plant pathogens [41,44,70].

In plants, the mitogen-activated protein kinase (MAPK) cascade plays a central role in the signal transduction of PAMP-triggered immunity (PTI) and Effector-Triggered Immunity (ETI) pathways, to activate defense-related genes and TFs: additionally, WRKY TFs play indispensable roles in this pathway. Consistent with the previous studies in *A. thaliana* and tomato plants infected with PSTVd [71,72], we observed the massive up-regulation of WRKY22 TFs, suggesting their involvement in the activation of the MAPK mediated defense signaling pathway in hop upon CBCVd infection. The intricate transcriptional regulation of stress-responsive genes is crucial to the plant’s defense response, and WRKY TFs act as the master regulators for several defense responsive genes [4]. In this context, we observed an extensive up-regulation of several WRKY TFs (WRKY40, 68, 72, 75 and 31) in the CBCVd-responsive transcriptome. Previous studies have shown the crucial role of these WRKY TFs in the basal defense in tomatoes and *A. thaliana* [73,74] or in the resistance to *Ralstonia solanacearum* in tobacco [75]; *Xanthomonas oryzae* in rice [76]; and *Phytophthora sojae* in soybeans [77]. Therefore, we strongly believe that the suite of WRKY TFs plays an indispensable role in triggering defense signaling cascades in response to CBCVd infection in the hop. In addition to the WRKY TFs, a strong up-regulation was observed for bZIP and NAC TFs families. The TGA7 (bZIP TF) transcription factors, which are implicated as regulators of pathogenesis-related (PR) genes in *A. thaliana* [78] and NAC78 (having an important role in defending against *Alternaria alternata* infecting Callery pears [79]) were found to be profoundly induced in our study.

The intricate network of phytohormone signaling pathways enables plants to activate appropriate and effective defense responses against pathogens by maintaining an appropriate balance between defense and growth [80]. Among other classes of phytohormones, salicylic acid (SA), jasmonic acid (JA), and ethylene (ET) are regarded as core immune phytohormones. The cross talk between phytohormone defense-signaling pathways are common and can be either mutually antagonistic or synergistic, resulting in negative or positive functional outcomes and thus providing the plant with a powerful regulatory potential. In line with the previous observation of the high up-regulation of genes involved in the SA and JA hormone signaling pathways in PSTVd infected tomato plants [69], we observed the significant up-regulation of TFs involved in these phytohormone signaling pathways in the CBCVd-infected hop. For instance, WRKY22 and 27 showed a significantly high expression, which is known to have dominant roles in the SA-JA cross talk during pathogen defense. The CaWRKY27 TF in pepper (*Capsicum annum*) positively regulates the stress resistance response to *R. solanacearum* infection through the modulation of SA, JA and ET-mediated signaling pathways in tobacco (*Nicotiana tabacum*) [81], whereas AtWRKY22 mediates the cross talk between SA and JA-dependent defense pathways against bacterial and fungal pathogens in *A. thaliana*. [82,83]. Similarly, ERF 109, which is shown to mediate the cross-talk between jasmonic acid and auxin biosynthesis in *A. thaliana* [84], was found to be up-regulated in our study. 

It is well known that a TF can act as an activator or repressor in the transcriptional regulation of gene expression [3]. Consistent with this observation, we found that 84 TF genes (18%) were down-regulated in our study, suggesting their role as a repressor during CBCVd infection in the hop. It is well established that when a plant perceives a pathogen, it switches to produce compounds necessary for a defense response by strategically compromising the mode of normal growth and development through the massive transcriptional regulation of gene expression via TFs [85,86], which was also evident from the results of the present study. Intriguingly, the SHINE and CUP-SHAPED COTYLEDON 3 TFs, which are involved in the regulation of flower organs [87] and leaf development [88] respectively in *A. thaliana*, were down-regulated in our study, suggesting their plausible roles in leaf deformation and cone size reduction upon CBCVd-infection in the hop.

In conclusion, our results provide a global overview of the role of TFs in mounting the defense response against CBCVd-infection in the hop. However, a functional analysis based on experimental approaches is essential for the screening of suitable TFs and their involvement in the transcriptional network in the defense response against CBCVd infection in the hop, which could be further utilized for genetic engineering and breeding programmes to improve crop resistance against viroid pathogens.

## Figures and Tables

**Table 1:** Details of the hop transcription factor (TFs) families identified in this study. The TFs were identified using a combination of the plant TF database (https://plantgrn.noble.org/PlantTFcat/) and HMMER searches using the assembled transcriptome sequences of hop plants infected with *Citrus bark cracking viroid* (CBCVd).

Transcription Factor Families	Count	Transcription Factor Families	Count	Transcription Factor Families	Count
A20-like	12	HD-ZIP	14	STY-LRP1	8
ABTB	5	HMG	12	SWIB-Plus-3	5
AP2-EREBP*	140	Homeodomain-LIKE	9	TAZ	2
ARF	20	Homeodomain-PHD	1	TCP	16
ARID	6	Homeodomain-TALE-BEL	11	Tc-PD	3
ARID-HMG	3	Homeodomain-TALE-KNOX	9	Tesmin	2
AS2-LOB	48	Homobox-WOX	82	TIFY	12
AUX-IAA	21	HSF-type-DNA-binding	19	TTF-type(Zn)	18
B3-Domain	48	ISWI	1	TUBBY	8
BED-type(Zn)	23	JmjC	20	WD40-like	276
BES/BZR	8	JmjC-ARID	1	WRKY*	66
bHLH*	106	JmjN	7	YEATS	3
Bromodomain	22	JUMONJI	7	ZF-HD	11
BTB-POZ	32	Lambda-DB	4	Znf-B	28
BTB-POZ-MATH	5	LFY	1	Znf-LSD	7
bZIP*	64	LIM	16	**Grand Total**	**3818**
C2C2-CO-like	27	LisH	21		
C2C2-Dof	26	MADS-MIKC	17		
C2C2-GATA	24	MADS-type1	30		
C2C2-YABBY	8	MYB*	16		
C2H2	428	MYB/SANT	24		
C3H	67	MYB-HB-like	218		
C3H-WRC/GRF	17	MYB-related	1		
CCHC(Zn)	893	NAC*	74		
CG1-CAMTA	5	Nin-like	6		
CHROMO-DOMAIN	114	NOZZLE	1		
CW-Zn	6	PAZ-Argonaute	11		
CW-Zn-B3/VAL	2	PHD	107		
DDT	7	PLATZ	13		
E2F-DP	7	RAV	4		
EIL	6	RB	1		
FAR	39	RR-A-type	31		
FHA-SMAD	19	RR-B-type	4		
FYR	6	S1Fa-like	1		
GAGA-Binding-like	4	SAP	9		
GARP-G2-like	6	SBP	23		
GeBP	2	SET	29		
GRAS	39	SNF2	41		
GRF	7	SPK	1		
Hap2/NF-YA	9	ssDNA-binding-TF	8		
Hap3/NF-YB	102	SSXT	2		
HD-SAD	12	STAT	1		

* TFs studied in this communication.

## Supplementary Materials

The following are available online at https://www.mdpi.com/1999-4915/11/5/419/s1, **Figure S1** Phylogenetic relationships and architecture of conserved protein motifs in hop TF genes. The phylogenetic tree was constructed based on the full-length sequences of hop TF proteins using MEGA 7 software. The motifs numbers 1–10, are displayed in different colored boxes and sequence information of the motifs are presented in the adjacent box. The sequence information for each motif is provided in the boxes. **Table S1**: List of qRT-PCR primers used for the validation of expression profiles of selected hop TFs. **Table S2**: Details of NCBI blast hits of CBCVd-responsive hop TFs. **Table S3**: Description of protein properties and predicted subcellular localization of CBCVd-responsive hop TFs. **Table S4**: Digital expression profiles of CBCVd-responsive hop TFs derived from FPKM values. **Table S5**: List of CBCVd-responsive hop TF orthologous genes in *Arabidopsis thaliana*. **Table S6**: Description of putative functions of proteins interacting with the CBCVd-responsive hop TFs identified in this study.

## References

[1] Moore J.W., Loake G.J., Spoel S.H. (2011). Transcription Dynamics in Plant Immunity. Plant Cell.

[2] Singh K.B., Foley R.C., Oñate-Sánchez L. (2002). Transcription factors in plant defense and stress responses. Curr. Opin. Plant Biol..

[3] Tsuda K., Somssich I.E. (2015). Tansley review Transcriptional networks in plant immunity. New Phytol..

[4] Eulgem T., Somssich I.E. (2007). Networks of WRKY transcription factors in defense signaling. Curr. Opin. Plant Biol..

[5] Stracke R., Werber M., Weisshaar B. (2001). The R2R3-MYB gene family in *Arabidopsis thaliana*. Curr. Opin. Plant Biol..

[6] Toledo-Ortiz G., Huq E., Quail P.H. (2003). The *Arabidopsis* Basic/Helix-Loop-Helix Transcription Factor Family. Plant Cell.

[7] Jakoby M., Weisshaar B., Dröge-Laser W., Vicente-Carbajosa J., Tiedemann J., Kroj T., Parcy F. (2002). bZIP transcription factors in *Arabidopsis*. Trends Plant Sci..

[8] Muthamilarasan M., Bonthala V.S., Mishra A.K., Khandelwal R., Khan Y., Roy R., Prasad M. (2014). C2H2 type of zinc finger transcription factors in foxtail millet define response to abiotic stresses. Funct. Integr. Genom..

[9] Muthamilarasan M., Bonthala V.S., Khandelwal R., Jaishankar J., Shweta S., Nawaz K., Prasad M. (2015). Global analysis of WRKY transcription factor superfamily in *Setaria* identifies potential candidates involved in abiotic stress signaling. Front. Plant Sci..

[10] Muthamilarasan M., Mangu V.R., Zandkarimi H., Prasad M., Baisakh N. (2016). Structure, organization and evolution of ADP-ribosylation factors in rice and *foxtail millet*, and their expression in rice. Sci. Rep..

[11] Saidi M.N., Mergby D., Brini F. (2017). Identification and expression analysis of the NAC transcription factor family in durum wheat (*Triticum turgidum* L. ssp. durum). Plant Physiol. Biochem..

[12] Yang J., Gao M., Huang L., Wang Y., van Nocker S., Wan R., Guo C., Wang X., Gao H. (2017). Identification and expression analysis of the apple (*Malus × domestica*) basic helix-loop-helix transcription factor family. Sci. Rep..

[13] Wen C.L., Cheng Q., Zhao L., Mao A., Yang J., Yu S., Weng Y., Xu Y. (2016). Identification and characterisation of Dof transcription factors in the cucumber genome. Sci. Rep..

[14] Hu W., Yang H., Yan Y., Wei Y., Tie W., Ding Z., Zuo J., Peng M., Li K. (2016). Genome-wide characterization and analysis of bZIP transcription factor gene family related to abiotic stress in cassava. Sci. Rep..

[15] Ding B. (2009). The Biology of Viroid-Host Interactions. Annu. Rev. Phytopathol..

[16] Hernández C., Flores R., de Alba A.E.M., Daròs J.-A., Serio F. (2005). Di Viroids and Viroid-Host Interactions. Annu. Rev. Phytopathol..

[17] Wilson C.R., Hay F.S., Eastwell K.C., Pethybridge S.J., Barbara D.J. (2008). Viruses and Viroids Infecting Hop: Significance, Epidemiology, and Management. Plant Dis..

[18] Pokorn T., Radišek S., Javornik B., Štajner N., Jakše J. (2017). Development of hop transcriptome to support research into host-viroid interactions. PLoS ONE.

[19] Jakse J., Radisek S., Pokorn T., Matousek J., Javornik B. (2015). Deep-sequencing revealed *Citrus bark cracking viroid* (CBCVd) as a highly aggressive pathogen on hop. Plant Pathol..

[20] Mishra A.K., Duraisamy G.S., Matoušek J., Radisek S., Javornik B., Jakse J. (2016). Identification and characterization of microRNAs in *Humulus lupulus* using high-throughput sequencing and their response to *Citrus bark cracking viroid* (CBCVd) infection. BMC Genomics.

[21] Mishra K.A., Kumar A., Mishra D., Nath S.V., Jakše J., Kocábek T., Killi K.U., Morina F., Matoušek J. (2018). Genome-Wide Transcriptomic Analysis Reveals Insights into the Response to *Citrus bark cracking viroid* (CBCVd) in Hop (*Humulus lupulus* L.). Viruses.

[22] Natsume S., Takagi H., Shiraishi A., Murata J., Toyonaga H., Patzak J., Takagi M., Yaegashi H., Uemura A., Mitsuoka C. (2015). The draft genome of hop (*Humulus lupulus*), an essence for brewing. Plant Cell Physiol..

[23] Dai X., Sinharoy S., Udvardi M., Zhao P.X. (2013). PlantTFcat: An online plant transcription factor and transcriptional regulator categorization and analysis tool. BMC Bioinform..

[24] Yu C.-S., Chen Y.-C., Lu C.-H., Hwang J.-K. (2006). Prediction of protein subcellular localization. Proteins Struct. Funct. Bioinforma..

[25] Conesa A., Götz S. (2008). Blast2GO: A Comprehensive Suite for Functional Analysis in Plant Genomics. Int. J. Plant Genom..

[26] Edgar R.C. (2004). MUSCLE: Multiple sequence alignment with high accuracy and high throughput. Nucleic Acids Res..

[27] Kumar S., Stecher G., Tamura K. (2016). MEGA7: Molecular Evolutionary Genetics Analysis Version 7.0 for Bigger Datasets. Mol. Biol. Evol..

[28] Haas B.J., Papanicolaou A., Yassour M., Grabherr M., Blood P.D., Bowden J., Couger M.B., Eccles D., Li B., Lieber M. (2013). De novo transcript sequence reconstruction from RNA-seq using the Trinity platform for reference generation and analysis. Nat. Protoc..

[29] Love M.I., Huber W., Anders S. (2014). Moderated estimation of fold change and dispersion for RNA-seq data with DESeq2. Genome Biol..

[30] Benjamini Y., Hochberg Y. (1995). Controlling the False Discovery Rate: A Practical and Powerful Approach to Multiple Testing. J. R. Stat. Soc. Ser. B.

[31] Metsalu T., Vilo J. (2015). ClustVis: A web tool for visualizing clustering of multivariate data using Principal Component Analysis and heatmap. Nucleic Acids Res..

[32] Huerta-Cepas J., Forslund K., Coelho L.P., Szklarczyk D., Jensen L.J., Von Mering C., Bork P. (2017). Fast genome-wide functional annotation through orthology assignment by eggNOG-mapper. Mol. Biol. Evol..

[33] Huerta-Cepas J., Szklarczyk D., Forslund K., Cook H., Heller D., Walter M.C., Rattei T., Mende D.R., Sunagawa S., Kuhn M. (2016). EGGNOG 4.5: A hierarchical orthology framework with improved functional annotations for eukaryotic, prokaryotic and viral sequences. Nucleic Acids Res..

[34] Morris J.H., Huerta-Cepas J., Junge A., Szklarczyk D., Jensen L.J., von Mering C., Lyon D., Gable A.L., Wyder S., Simonovic M. (2018). STRING v11: Protein–protein association networks with increased coverage, supporting functional discovery in genome-wide experimental datasets. Nucleic Acids Res..

[35] Chin C.-H., Chen S.-H., Wu H.-H., Ho C.-W., Ko M.-T., Lin C.-Y. (2014). cytoHubba: Identifying hub objects and sub-networks from complex interactome. BMC Syst. Biol..

[36] Shannon P., Markiel A., Ozier O., Baliga N.S., Wang J.T., Ramage D., Amin N., Schwikowski B., Ideker T. (2003). Cytoscape: A software environment for integrated models of biomolecular interaction networks. Genome Res..

[37] Marshall O.J. (2004). PerlPrimer: Cross-platform, graphical primer design for standard, bisulphite and real-time PCR. Bioinformatics.

[38] Ye J., Coulouris G., Zaretskaya I., Cutcutache I., Rozen S., Madden T.L. (2012). Primer-BLAST: A tool to design target-specific primers for polymerase chain reaction. BMC Bioinform..

[39] Livak K.J., Schmittgen T.D. (2001). Analysis of relative gene expression data using real-time quantitative PCR and the 2(-Delta Delta C(T)) Method. Methods.

[40] Štajner N., Cregeen S., Javornik B. (2013). Evaluation of reference genes for RT-qPCR expression studies in hop (*Humulus lupulus* L.) during infection with vascular pathogen verticillium albo-atrum. PLoS ONE.

[41] McGrath K.C., Dombrecht B., Manners J.M., Schenk P.M., Edgar C.I., Maclean D.J., Scheible W.-R., Udvardi M.K., Kazan K. (2005). Repressor- and Activator-Type Ethylene Response Factors Functioning in Jasmonate Signaling and Disease Resistance Identified via a Genome-Wide Screen of *Arabidopsis* Transcription Factor Gene Expression. Plant Physiol..

[42] Seo E., Choi D. (2015). Functional studies of transcription factors involved in plant defenses in the genomics era. Brief. Funct. Genom..

[43] Sakuma Y., Liu Q., Dubouzet J.G., Abe H., Shinozaki K., Yamaguchi- Shinozaki K. (2002). DNA-binding specificity of the ERF/AP2 domain of Arabidopsis DREBs, transcription factors involved in dehydration- and cold-inducible gene expression. Biochem. Biophys. Res. Commun..

[44] Eulgem T. (2005). Regulation of the *Arabidopsis* defense transcriptome. Trends Plant Sci..

[45] Zhu T., Nevo E., Sun D., Peng J. (2012). Phylogenetic analyses unravel the evolutionary history of nac proteins in plants. Evolution.

[46] Buscaill P., Rivas S. (2014). Transcriptional control of plant defence responses. Curr. Opin. Plant Biol..

[47] Riechmann J.L., Heard J., Martin G., Reuber L., Jiang C.-Z., Keddie J., Adam L., Pineda O., Ratcliffe O.J., Samaha R.R. (2000). Transcription Factors: Genome-Wide Comparative Analysis Among Eukaryotes. Science.

[48] Nakano T., Suzuki K., Fujimura T., Shinshi H. (2006). Genome-Wide Analysis of the ERF Gene Family in Arabidopsis and Rice. Plant Physiol..

[49] Fan K., Wang M., Miao Y., Ni M., Bibi N., Yuan S., Li F., Wang X. (2014). Molecular evolution and expansion analysis of the NAC transcription factor in Zea mays. PLoS ONE.

[50] Shiu S.-H., Shih M.-C., Li W.-H. (2005). Transcription factor families have much higher expansion rates in plants than in animals. Plant Physiol..

[51] Wen J., Zhang J.-Q., Nie Z.-L., Zhong Y., Sun H. (2014). Evolutionary diversifications of plants on the Qinghai-Tibetan Plateau. Front. Genet..

[52] Kaessmann H. (2010). Origins, evolution, and phenotypic impact of new genes. Genome Res..

[53] Muthamilarasan M., Khandelwal R., Yadav C.B., Bonthala V.S., Khan Y., Prasad M. (2014). Identification and molecular characterization of MYB Transcription Factor Superfamily in C4 model plant foxtail millet (*Setaria italica* L.). PLoS ONE.

[54] Tompa P., Davey N.E., Gibson T.J., Babu M.M. (2014). A Million peptide motifs for the molecular biologist. Mol. Cell.

[55] Wu K., Wu K., Guo Z., Guo Z., Wang H., Wang H., Li J., Li J. (2005). The WRKY Family of Transcription Factors in Rice and. Gene.

[56] Xie T., Chen C., Li C., Liu J., Liu C., He Y. (2018). Genome-wide investigation of WRKY gene family in pineapple: Evolution and expression profiles during development and stress. BMC Genom..

[57] Nath V.S., Koyyappurath S., Alex T.E., Geetha K.A., Augustine L., Nasser A., Thomas G. (2018). Transcriptome-based mining and expression profiling of *Pythium* responsive transcription factors in Zingiber sp. Funct. Integr. Genom..

[58] Tatusov R.L., Koonin E.V., Lipman D.J. (1997). A genomic perspective on genomic families. Science.

[59] Thornton J.W., DeSalle R. (2000). Gene Family Evolution and Homology: Genomics Meets Phylogenetics. Annu. Rev. Genom. Hum. Genet..

[60] Chen R., Jeong S.S. (2000). Functional prediction: Identification of protein orthologs and paralogs. Protein Sci..

[61] Jiang Z., Dong X., Zhang Z. (2016). Network-Based Comparative Analysis of *Arabidopsis* Immune Responses to Golovinomyces orontii and *Botrytis cinerea* Infections. Sci. Rep..

[62] Reményi A., Good M.C., Bhattacharyya R.P., Lim W.A. (2005). The Role of Docking Interactions in Mediating Signaling Input, Output, and Discrimination in the Yeast MAPK Network. Mol. Cell.

[63] Alves M.S., Dadalto S.P., Gonçalves A.B., de Souza G.B., Barros V.A., Fietto L.G. (2014). Transcription Factor Functional Protein-Protein Interactions in Plant Defense Responses. Proteomes.

[64] Geisler-Lee J., O’Toole N., Ammar R., Provart N.J., Millar A.H., Geisler M. (2007). A predicted interactome for Arabidopsis. Plant Physiol..

[65] Musungu B., Bhatnagar D., Brown R.L., Fakhoury A.M., Geisler M. (2015). A predicted protein interactome identifies conserved global networks and disease resistance subnetworks in maize. Front. Genet..

[66] Chi Y., Yang Y., Zhou Y., Zhou J., Fan B., Yu J.-Q., Chen Z. (2013). Protein–Protein Interactions in the Regulation of WRKY Transcription Factors. Mol. Plant.

[67] Owens R.A., Tech K.B., Shao J.Y., Sano T., Baker C.J. (2012). Global Analysis of Tomato Gene Expression During Potato spindle tuber viroid Infection Reveals a Complex Array of Changes Affecting Hormone Signaling. Mol. Plant-Microbe Interact..

[68] Katsarou K., Wu Y., Zhang R., Bonar N., Morris J., Hedley P.E., Bryan G.J., Kalantidis K., Hornyik C. (2016). Insight on Genes Affecting Tuber Development in Potato upon Potato spindle tuber viroid (PSTVd) Infection. PLoS One.

[69] Więsyk A., Iwanicka-Nowicka R., Fogtman A., Zagórski-Ostoja W., Góra-Sochacka A. (2018). Time-Course Microarray Analysis Reveals Differences between Transcriptional Changes in Tomato Leaves Triggered by Mild and Severe Variants of Potato Spindle Tuber Viroid. Viruses.

[70] Dubos C., Stracke R., Grotewold E., Weisshaar B., Martin C., Lepiniec L. (2010). MYB transcription factors in Arabidopsis. Trends Plant Sci..

[71] Asai T., Tena G., Plotnikova J., Willmann M.R., Chiu W.-L., Gomez-Gomez L., Boller T., Ausubel F.M., Sheen J. (2002). MAP kinase signalling cascade in *Arabidopsis* innate immunity. Nature.

[72] Zheng Y., Wang Y., Ding B., Fei Z. (2017). Comprehensive Transcriptome Analyses Reveal that *Potato Spindle Tuber Viroid* Triggers Genome-Wide Changes in Alternative Splicing, Inducible trans-Acting Activity of Phased Secondary Small Interfering RNAs, and Immune Responses. J. Virol..

[73] Maldonado A.M., Amil-Ruiz F., Muñoz-Blanco J., Encinas-Villarejo S., Caballero J.L., de los Santos B., Romero F., Pliego-Alfaro F. (2009). Evidence for a positive regulatory role of strawberry (Fragaria×ananassa) Fa WRKY1 and Arabidopsis At WRKY75 proteins in resistance. J. Exp. Bot..

[74] Bhattarai K.K., Atamian H.S., Kaloshian I., Eulgem T. (2010). WRKY72-type transcription factors contribute to basal immunity in tomato and *Arabidopsis* as well as gene-for-gene resistance mediated by the tomato R gene Mi-1. Plant J..

[75] Shi W., Hao L., Li J., Liu D., Guo X., Li H. (2014). The *Gossypium hirsutum* WRKY gene GhWRKY39-1 promotes pathogen infection defense responses and mediates salt stress tolerance in transgenic *Nicotiana benthamiana*. Plant Cell Rep..

[76] YANG S., ZHOU L., MIAO L., SHI J., SUN C., FAN W., LAN J., CHEN H., LIU L., DOU S. (2016). The expression and binding properties of the rice WRKY68 protein in the Xa21-mediated resistance response to *Xanthomonas oryzae* pv. Oryzae. J. Integr. Agric..

[77] Fan S., Dong L., Han D., Zhang F., Wu J., Jiang L., Cheng Q., Li R., Lu W., Meng F. (2017). GmWRKY31 and GmHDL56 Enhances Resistance to *Phytophthora sojae* by Regulating Defense-Related Gene Expression in Soybean. Front. Plant Sci..

[78] Shearer H.L., Wang L., DeLong C., Despres C., Fobert P.R. (2009). NPR1 enhances the DNA binding activity of the *Arabidopsis* bZIP transcription factor TGA7This paper is one of a selection of papers published in a Special Issue from the National Research Council of Canada – Plant Biotechnology Institute. Botany.

[79] Kan J., Liu T., Ma N., Li H., Li X., Wang J., Zhang B., Chang Y., Lin J. (2017). Transcriptome analysis of Callery pear (*Pyrus calleryana*) reveals a comprehensive signalling network in response to *Alternaria alternata*. PLoS ONE.

[80] Berens M.L., Berry H.M., Mine A., Argueso C.T., Tsuda K. (2017). Evolution of Hormone Signaling Networks in Plant Defense. Annu. Rev. Phytopathol..

[81] Dang F., Wang Y., She J., Lei Y., Liu Z., Eulgem T., Lai Y., Lin J., Yu L., Lei D. (2014). Overexpression of CaWRKY27, a subgroup IIe WRKY transcription factor of *Capsicum annuum*, positively regulates tobacco resistance to Ralstonia solanacearum infection. Physiol. Plant..

[82] Li J., Brader G., Kariola T., Tapio Palva E. (2006). WRKY70 modulates the selection of signaling pathways in plant defense. Plant J..

[83] Phukan U.J., Jeena G.S., Shukla R.K. (2016). WRKY Transcription Factors: Molecular Regulation and Stress Responses in Plants. Front. Plant Sci..

[84] Cai X.-T., Xu P., Zhao P.-X., Liu R., Yu L.-H., Xiang C.-B. (2014). *Arabidopsis* ERF109 mediates cross-talk between jasmonic acid and auxin biosynthesis during lateral root formation. Nat. Commun..

[85] Scheideler M., Schlaich N.L., Fellenberg K., Beissbarth T., Hauser N.C., Vingron M., Slusarenko A.J., Hoheisel J.D. (2002). Monitoring the Switch from Housekeeping to Pathogen Defense Metabolism in *Arabidopsis thaliana* Using cDNA Arrays. J. Biol. Chem..

[86] Windram O., Madhou P., McHattie S., Hill C., Hickman R., Cooke E., Jenkins D.J., Penfold C.A., Baxter L., Breeze E. (2012). *Arabidopsis* Defense against *Botrytis cinerea*: Chronology and Regulation Deciphered by High-Resolution Temporal Transcriptomic Analysis. Plant Cell.

[87] Shi J.X., Malitsky S., de Oliveira S., Branigan C., Franke R.B., Schreiber L., Aharoni A. (2011). SHINE transcription factors act redundantly to pattern the archetypal surface of *arabidopsis* flower organs. PLoS Genet..

[88] Hasson A., Plessis A., Blein T., Adroher B., Grigg S., Tsiantis M., Boudaoud A., Damerval C., Laufs P. (2011). Evolution and Diverse Roles of the CUP-SHAPED COTYLEDON Genes in *Arabidopsis* Leaf Development. Plant Cell.

