# Supplementary material for: Revisiting the Role of Transcription Factors in Coordinating the Defense Response Against Citrus Bark Cracking Viroid Infection in Commercial Hop (Humulus Lupulus L.)
Source: Viruses. 2019 May 5;11(5):419. doi: 10.3390/v11050419 (PMC6563305; doi:10.3390/v11050419)
Supplement: Supplementary file 1 [file viruses-11-00419-s001.zip › Supplementary Fig. 1.pdf]

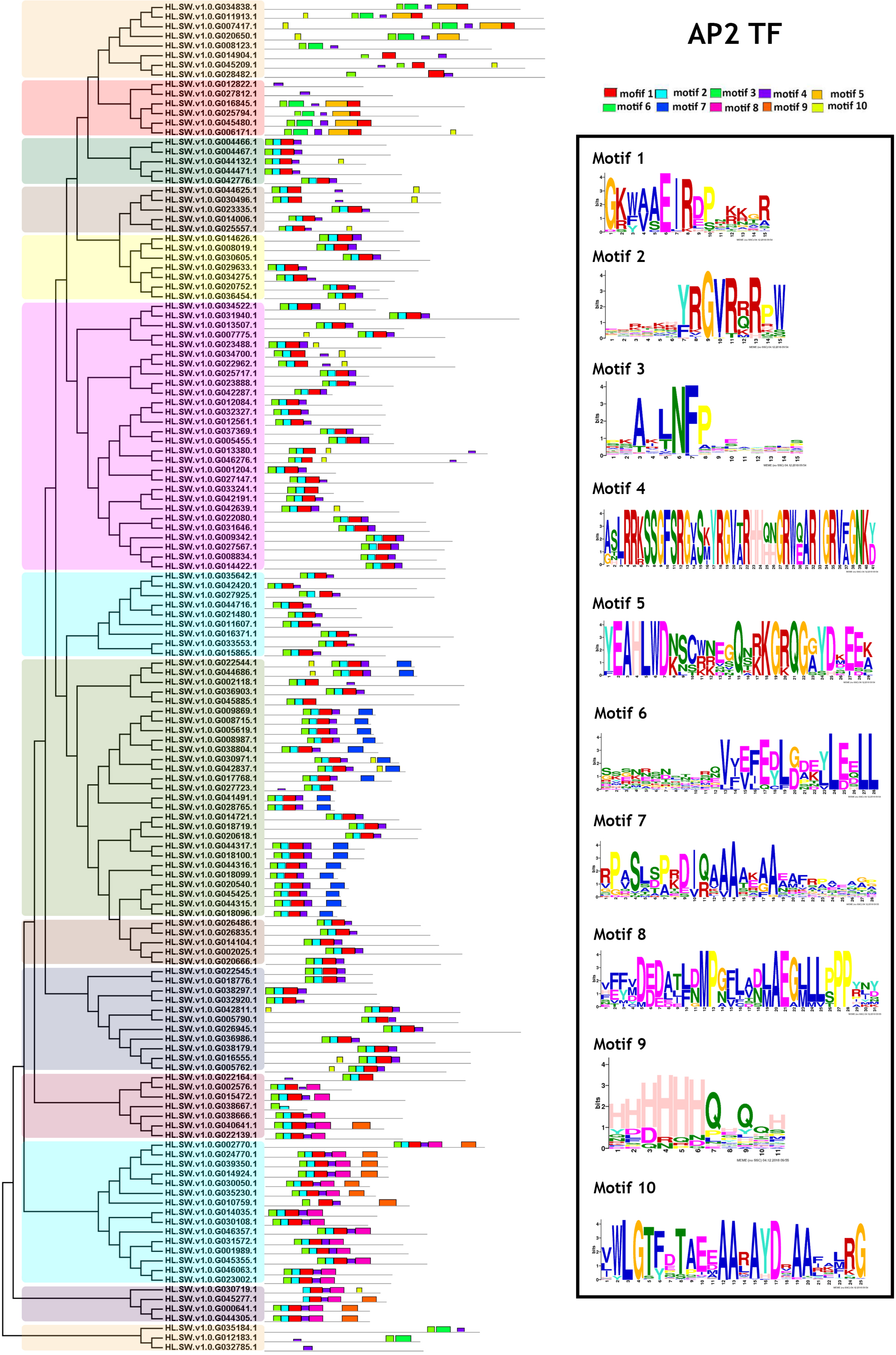

AP2 TF
motif 1
motif 2
motif 3
motif 4
motif 5
motif 6
motif 7
motif 8
motif 9
motif 10
Motif 1
Motif 2
Motif 3
Motif 4
Motif 5
Motif 6
Motif 7
Motif 8
Motif 9
Motif 10
HL.SW.v1.0.G034838.1
HL.SW.v1.0.G011913.1
HL.SW.v1.0.G007417.1
HL.SW.v1.0.G020650.1
HL.SW.v1.0.G008123.1
HL.SW.v1.0.G014904.1
HL.SW.v1.0.G045209.1
HL.SW.v1.0.G028482.1
HL.SW.v1.0.G012822.1
HL.SW.v1.0.G027812.1
HL.SW.v1.0.G016845.1
HL.SW.v1.0.G025794.1
HL.SW.v1.0.G045480.1
HL.SW.v1.0.G006171.1
HL.SW.v1.0.G004466.1
HL.SW.v1.0.G004467.1
HL.SW.v1.0.G044132.1
HL.SW.v1.0.G044471.1
HL.SW.v1.0.G042776.1
HL.SW.v1.0.G044625.1
HL.SW.v1.0.G030496.1
HL.SW.v1.0.G023335.1
HL.SW.v1.0.G014006.1
HL.SW.v1.0.G025557.1
HL.SW.v1.0.G014626.1
HL.SW.v1.0.G008019.1
HL.SW.v1.0.G030605.1
HL.SW.v1.0.G029633.1
HL.SW.v1.0.G034275.1
HL.SW.v1.0.G020752.1
HL.SW.v1.0.G036454.1
HL.SW.v1.0.G034522.1
HL.SW.v1.0.G031940.1
HL.SW.v1.0.G013507.1
HL.SW.v1.0.G007775.1
HL.SW.v1.0.G023488.1
HL.SW.v1.0.G034700.1
HL.SW.v1.0.G022962.1
HL.SW.v1.0.G025717.1
HL.SW.v1.0.G023888.1
HL.SW.v1.0.G042287.1
HL.SW.v1.0.G012084.1
HL.SW.v1.0.G032327.1
HL.SW.v1.0.G012561.1
HL.SW.v1.0.G037369.1
HL.SW.v1.0.G005455.1
HL.SW.v1.0.G013380.1
HL.SW.v1.0.G046276.1
HL.SW.v1.0.G001204.1
HL.SW.v1.0.G027147.1
HL.SW.v1.0.G033241.1
HL.SW.v1.0.G042191.1
HL.SW.v1.0.G042639.1
HL.SW.v1.0.G022080.1
HL.SW.v1.0.G031646.1
HL.SW.v1.0.G009342.1
HL.SW.v1.0.G027567.1
HL.SW.v1.0.G008834.1
HL.SW.v1.0.G014422.1
HL.SW.v1.0.G035642.1
HL.SW.v1.0.G042420.1
HL.SW.v1.0.G027925.1
HL.SW.v1.0.G044716.1
HL.SW.v1.0.G021480.1
HL.SW.v1.0.G011607.1
HL.SW.v1.0.G016371.1
HL.SW.v1.0.G033553.1
HL.SW.v1.0.G015865.1
HL.SW.v1.0.G022544.1
HL.SW.v1.0.G044686.1
HL.SW.v1.0.G002118.1
HL.SW.v1.0.G036903.1
HL.SW.v1.0.G045885.1
HL.SW.v1.0.G009869.1
HL.SW.v1.0.G008715.1
HL.SW.v1.0.G005619.1
HL.SW.v1.0.G008987.1
HL.SW.v1.0.G038804.1
HL.SW.v1.0.G030971.1
HL.SW.v1.0.G042837.1
HL.SW.v1.0.G017768.1
HL.SW.v1.0.G027723.1
HL.SW.v1.0.G041491.1
HL.SW.v1.0.G028765.1
HL.SW.v1.0.G014721.1
HL.SW.v1.0.G018719.1
HL.SW.v1.0.G020618.1
HL.SW.v1.0.G044317.1
HL.SW.v1.0.G018100.1
HL.SW.v1.0.G044316.1
HL.SW.v1.0.G018099.1
HL.SW.v1.0.G020540.1
HL.SW.v1.0.G045425.1
HL.SW.v1.0.G044315.1
HL.SW.v1.0.G018096.1
HL.SW.v1.0.G026486.1
HL.SW.v1.0.G026835.1
HL.SW.v1.0.G014104.1
HL.SW.v1.0.G002025.1
HL.SW.v1.0.G020666.1
HL.SW.v1.0.G022545.1
HL.SW.v1.0.G018776.1
HL.SW.v1.0.G038297.1
HL.SW.v1.0.G032920.1
HL.SW.v1.0.G042811.1
HL.SW.v1.0.G005790.1
HL.SW.v1.0.G026945.1
HL.SW.v1.0.G036986.1
HL.SW.v1.0.G038179.1
HL.SW.v1.0.G016555.1
HL.SW.v1.0.G005762.1
HL.SW.v1.0.G022164.1
HL.SW.v1.0.G002576.1
HL.SW.v1.0.G015472.1
HL.SW.v1.0.G038667.1
HL.SW.v1.0.G038666.1
HL.SW.v1.0.G040641.1
HL.SW.v1.0.G022139.1
HL.SW.v1.0.G002770.1
HL.SW.v1.0.G024770.1
HL.SW.v1.0.G039350.1
HL.SW.v1.0.G014924.1
HL.SW.v1.0.G030050.1
HL.SW.v1.0.G035230.1
HL.SW.v1.0.G010759.1
HL.SW.v1.0.G014035.1
HL.SW.v1.0.G030108.1
HL.SW.v1.0.G046357.1
HL.SW.v1.0.G031572.1
HL.SW.v1.0.G001989.1
HL.SW.v1.0.G045355.1
HL.SW.v1.0.G046063.1
HL.SW.v1.0.G023002.1
HL.SW.v1.0.G030719.1
HL.SW.v1.0.G045277.1
HL.SW.v1.0.G000641.1
HL.SW.v1.0.G044305.1
HL.SW.v1.0.G035184.1
HL.SW.v1.0.G012183.1
HL.SW.v1.0.G032785.1

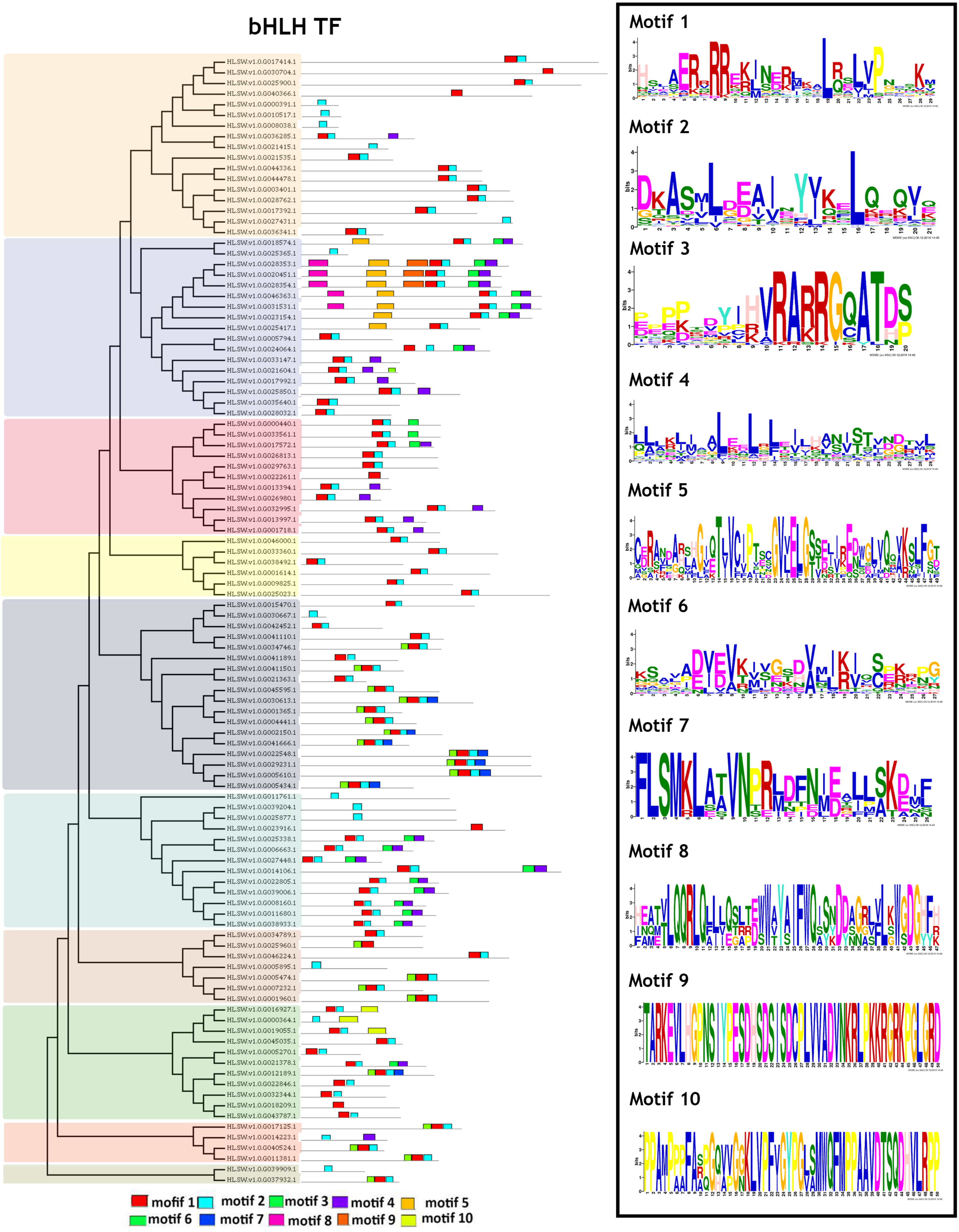
bHLH TF
HL.SW.v1.0.G017414.1
HL.SW.v1.0.G030704.1
HL.SW.v1.0.G025900.1
HL.SW.v1.0.G040366.1
HL.SW.v1.0.G000391.1
HL.SW.v1.0.G010517.1
HL.SW.v1.0.G008038.1
HL.SW.v1.0.G036285.1
HL.SW.v1.0.G021415.1
HL.SW.v1.0.G021535.1
HL.SW.v1.0.G044336.1
HL.SW.v1.0.G044478.1
HL.SW.v1.0.G003401.1
HL.SW.v1.0.G028762.1
HL.SW.v1.0.G017392.1
HL.SW.v1.0.G027431.1
HL.SW.v1.0.G036341.1
HL.SW.v1.0.G018574.1
HL.SW.v1.0.G025365.1
HL.SW.v1.0.G028353.1
HL.SW.v1.0.G020451.1
HL.SW.v1.0.G028354.1
HL.SW.v1.0.G046363.1
HL.SW.v1.0.G031531.1
HL.SW.v1.0.G023154.1
HL.SW.v1.0.G025417.1
HL.SW.v1.0.G005794.1
HL.SW.v1.0.G024064.1
HL.SW.v1.0.G033147.1
HL.SW.v1.0.G021604.1
HL.SW.v1.0.G017992.1
HL.SW.v1.0.G025850.1
HL.SW.v1.0.G035640.1
HL.SW.v1.0.G028032.1
HL.SW.v1.0.G000440.1
HL.SW.v1.0.G033561.1
HL.SW.v1.0.G017572.1
HL.SW.v1.0.G026813.1
HL.SW.v1.0.G029763.1
HL.SW.v1.0.G022261.1
HL.SW.v1.0.G013394.1
HL.SW.v1.0.G026980.1
HL.SW.v1.0.G032995.1
HL.SW.v1.0.G013997.1
HL.SW.v1.0.G001718.1
HL.SW.v1.0.G046000.1
HL.SW.v1.0.G033360.1
HL.SW.v1.0.G038492.1
HL.SW.v1.0.G001614.1
HL.SW.v1.0.G009825.1
HL.SW.v1.0.G025023.1
HL.SW.v1.0.G015470.1
HL.SW.v1.0.G030667.1
HL.SW.v1.0.G042452.1
HL.SW.v1.0.G041110.1
HL.SW.v1.0.G034746.1
HL.SW.v1.0.G041189.1
HL.SW.v1.0.G041150.1
HL.SW.v1.0.G021363.1
HL.SW.v1.0.G045595.1
HL.SW.v1.0.G030613.1
HL.SW.v1.0.G001365.1
HL.SW.v1.0.G004441.1
HL.SW.v1.0.G002150.1
HL.SW.v1.0.G041666.1
HL.SW.v1.0.G022548.1
HL.SW.v1.0.G029231.1
HL.SW.v1.0.G005610.1
HL.SW.v1.0.G005434.1
HL.SW.v1.0.G011761.1
HL.SW.v1.0.G039204.1
HL.SW.v1.0.G025877.1
HL.SW.v1.0.G023916.1
HL.SW.v1.0.G025338.1
HL.SW.v1.0.G006663.1
HL.SW.v1.0.G027448.1
HL.SW.v1.0.G014106.1
HL.SW.v1.0.G022805.1
HL.SW.v1.0.G039006.1
HL.SW.v1.0.G008160.1
HL.SW.v1.0.G011680.1
HL.SW.v1.0.G038933.1
HL.SW.v1.0.G034789.1
HL.SW.v1.0.G025960.1
HL.SW.v1.0.G046224.1
HL.SW.v1.0.G005895.1
HL.SW.v1.0.G005474.1
HL.SW.v1.0.G007232.1
HL.SW.v1.0.G001960.1
HL.SW.v1.0.G016927.1
HL.SW.v1.0.G000364.1
HL.SW.v1.0.G019055.1
HL.SW.v1.0.G045035.1
HL.SW.v1.0.G005270.1
HL.SW.v1.0.G021378.1
HL.SW.v1.0.G012189.1
HL.SW.v1.0.G022846.1
HL.SW.v1.0.G032344.1
HL.SW.v1.0.G018209.1
HL.SW.v1.0.G043787.1
HL.SW.v1.0.G017125.1
HL.SW.v1.0.G014223.1
HL.SW.v1.0.G040524.1
HL.SW.v1.0.G011381.1
HL.SW.v1.0.G039909.1
HL.SW.v1.0.G037932.1
motif 1
motif 2
motif 3
motif 4
motif 5
motif 6
motif 7
motif 8
motif 9
motif 10
Motif 1
Motif 2
Motif 3
Motif 4
Motif 5
Motif 6
Motif 7
Motif 8
Motif 9
Motif 10

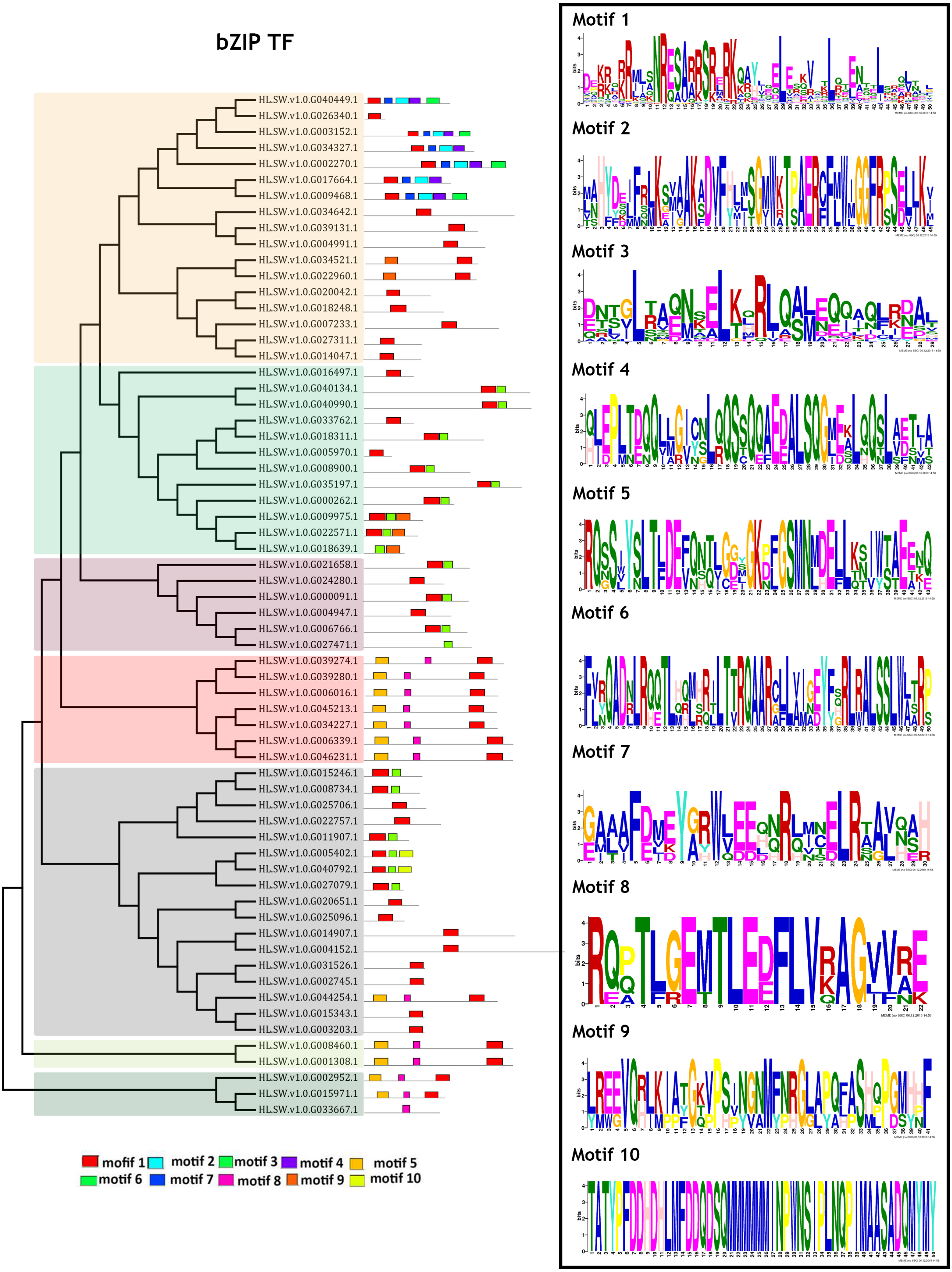
bZIP TF
HL.SW.v1.0.G040449.1
HL.SW.v1.0.G026340.1
HL.SW.v1.0.G003152.1
HL.SW.v1.0.G034327.1
HL.SW.v1.0.G002270.1
HL.SW.v1.0.G017664.1
HL.SW.v1.0.G009468.1
HL.SW.v1.0.G034642.1
HL.SW.v1.0.G039131.1
HL.SW.v1.0.G004991.1
HL.SW.v1.0.G034521.1
HL.SW.v1.0.G022960.1
HL.SW.v1.0.G020042.1
HL.SW.v1.0.G018248.1
HL.SW.v1.0.G007233.1
HL.SW.v1.0.G027311.1
HL.SW.v1.0.G014047.1
HL.SW.v1.0.G016497.1
HL.SW.v1.0.G040134.1
HL.SW.v1.0.G040990.1
HL.SW.v1.0.G033762.1
HL.SW.v1.0.G018311.1
HL.SW.v1.0.G005970.1
HL.SW.v1.0.G008900.1
HL.SW.v1.0.G035197.1
HL.SW.v1.0.G000262.1
HL.SW.v1.0.G009975.1
HL.SW.v1.0.G022571.1
HL.SW.v1.0.G018639.1
HL.SW.v1.0.G021658.1
HL.SW.v1.0.G024280.1
HL.SW.v1.0.G000091.1
HL.SW.v1.0.G004947.1
HL.SW.v1.0.G006766.1
HL.SW.v1.0.G027471.1
HL.SW.v1.0.G039274.1
HL.SW.v1.0.G039280.1
HL.SW.v1.0.G006016.1
HL.SW.v1.0.G045213.1
HL.SW.v1.0.G034227.1
HL.SW.v1.0.G006339.1
HL.SW.v1.0.G046231.1
HL.SW.v1.0.G015246.1
HL.SW.v1.0.G008734.1
HL.SW.v1.0.G025706.1
HL.SW.v1.0.G022757.1
HL.SW.v1.0.G011907.1
HL.SW.v1.0.G005402.1
HL.SW.v1.0.G040792.1
HL.SW.v1.0.G027079.1
HL.SW.v1.0.G020651.1
HL.SW.v1.0.G025096.1
HL.SW.v1.0.G014907.1
HL.SW.v1.0.G004152.1
HL.SW.v1.0.G031526.1
HL.SW.v1.0.G002745.1
HL.SW.v1.0.G044254.1
HL.SW.v1.0.G015343.1
HL.SW.v1.0.G003203.1
HL.SW.v1.0.G008460.1
HL.SW.v1.0.G001308.1
HL.SW.v1.0.G002952.1
HL.SW.v1.0.G015971.1
HL.SW.v1.0.G033667.1
motif 1
motif 2
motif 3
motif 4
motif 5
motif 6
motif 7
motif 8
motif 9
motif 10
Motif 1
Motif 2
Motif 3
Motif 4
Motif 5
Motif 6
Motif 7
Motif 8
Motif 9
Motif 10

MYB TF

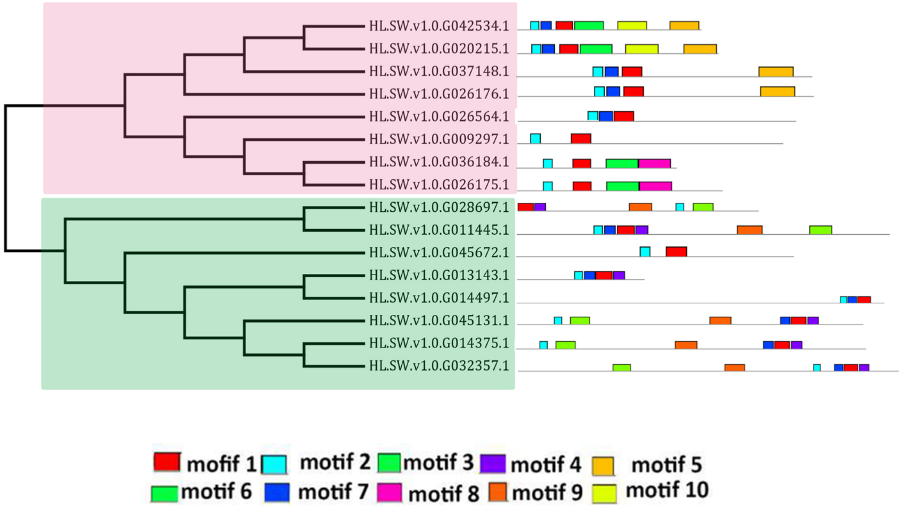
HL.SW.v1.0.G042534.1
HL.SW.v1.0.G020215.1
HL.SW.v1.0.G037148.1
HL.SW.v1.0.G026176.1
HL.SW.v1.0.G026564.1
HL.SW.v1.0.G009297.1
HL.SW.v1.0.G036184.1
HL.SW.v1.0.G026175.1
HL.SW.v1.0.G028697.1
HL.SW.v1.0.G011445.1
HL.SW.v1.0.G045672.1
HL.SW.v1.0.G013143.1
HL.SW.v1.0.G014497.1
HL.SW.v1.0.G045131.1
HL.SW.v1.0.G014375.1
HL.SW.v1.0.G032357.1
motif 1
motif 2
motif 3
motif 4
motif 5
motif 6
motif 7
motif 8
motif 9
motif 10

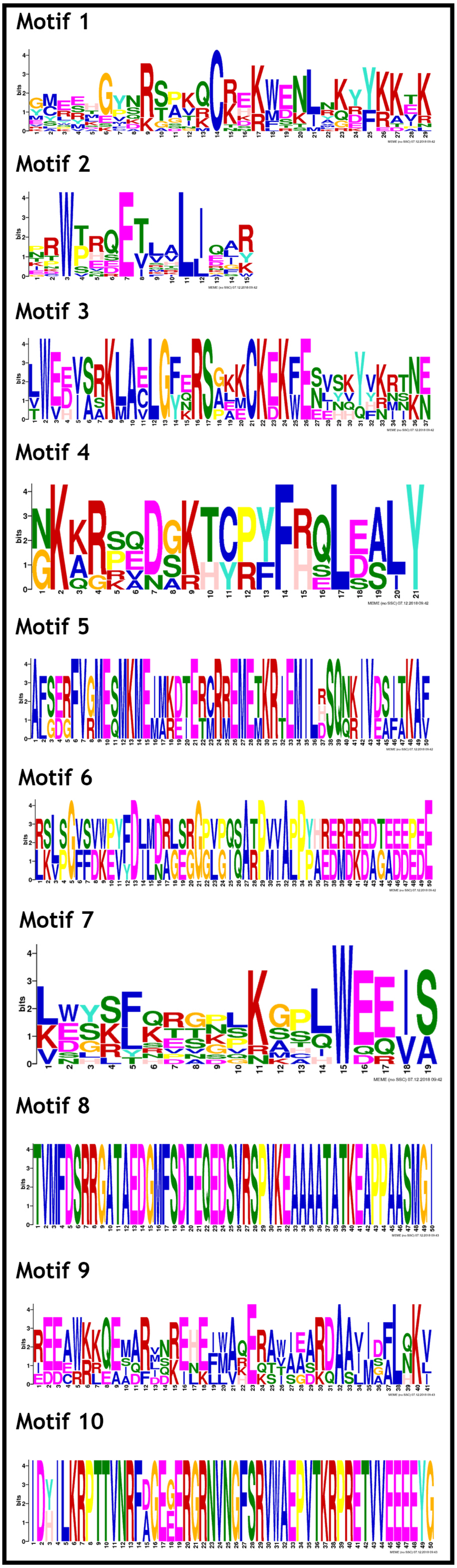
Motif 1
Motif 2
Motif 3
Motif 4
Motif 5
Motif 6
Motif 7
Motif 8
Motif 9
Motif 10

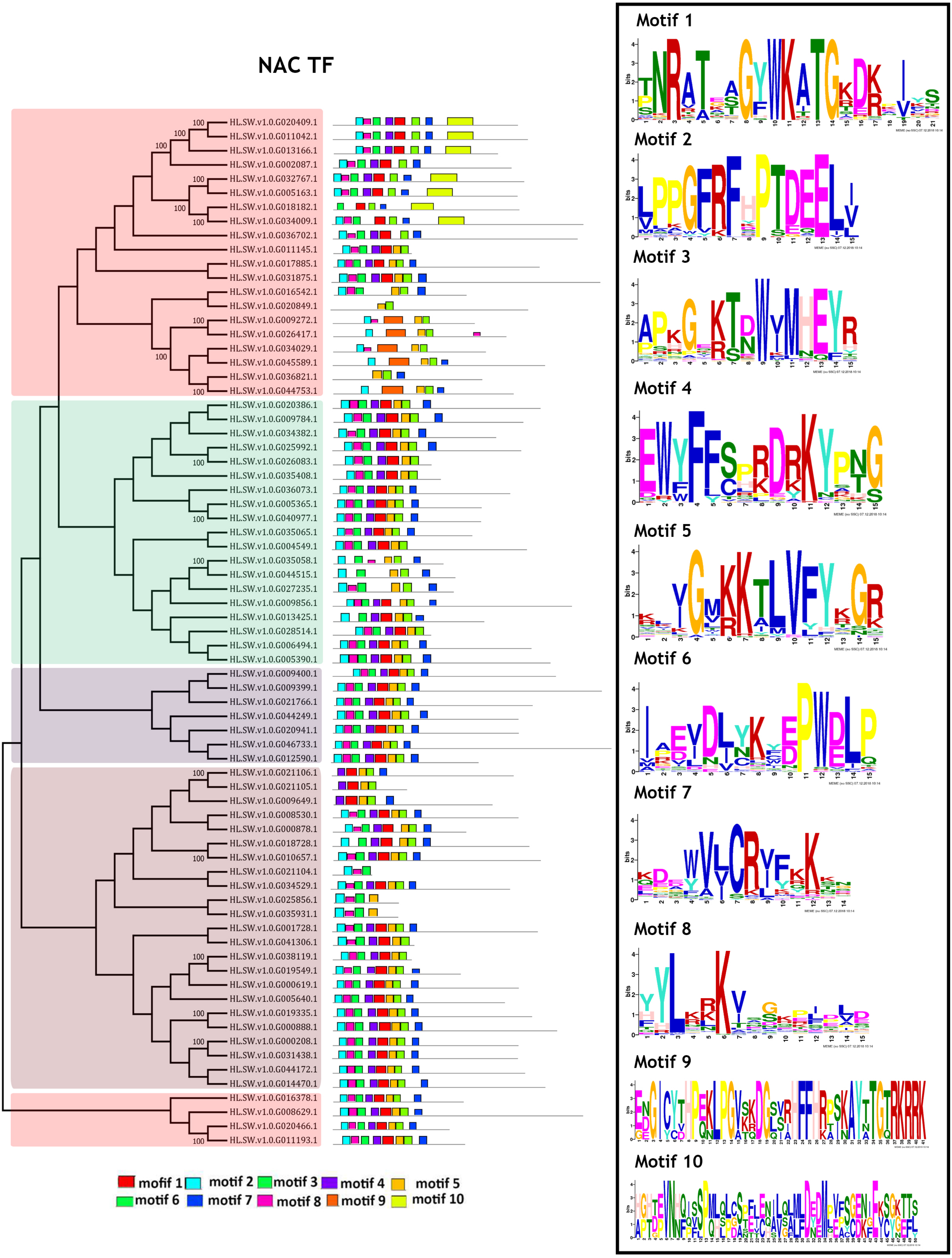
NAC TF
HL.SW.v1.0.G020409.1
HL.SW.v1.0.G011042.1
HL.SW.v1.0.G013166.1
HL.SW.v1.0.G002087.1
HL.SW.v1.0.G032767.1
HL.SW.v1.0.G005163.1
HL.SW.v1.0.G018182.1
HL.SW.v1.0.G034009.1
HL.SW.v1.0.G036702.1
HL.SW.v1.0.G011145.1
HL.SW.v1.0.G017885.1
HL.SW.v1.0.G031875.1
HL.SW.v1.0.G016542.1
HL.SW.v1.0.G020849.1
HL.SW.v1.0.G009272.1
HL.SW.v1.0.G026417.1
HL.SW.v1.0.G034029.1
HL.SW.v1.0.G045589.1
HL.SW.v1.0.G036821.1
HL.SW.v1.0.G044753.1
HL.SW.v1.0.G020386.1
HL.SW.v1.0.G009784.1
HL.SW.v1.0.G034382.1
HL.SW.v1.0.G025992.1
HL.SW.v1.0.G026083.1
HL.SW.v1.0.G035408.1
HL.SW.v1.0.G036073.1
HL.SW.v1.0.G005365.1
HL.SW.v1.0.G040977.1
HL.SW.v1.0.G035065.1
HL.SW.v1.0.G004549.1
HL.SW.v1.0.G035058.1
HL.SW.v1.0.G044515.1
HL.SW.v1.0.G027235.1
HL.SW.v1.0.G009856.1
HL.SW.v1.0.G013425.1
HL.SW.v1.0.G028514.1
HL.SW.v1.0.G006494.1
HL.SW.v1.0.G005390.1
HL.SW.v1.0.G009400.1
HL.SW.v1.0.G009399.1
HL.SW.v1.0.G021766.1
HL.SW.v1.0.G044249.1
HL.SW.v1.0.G020941.1
HL.SW.v1.0.G046733.1
HL.SW.v1.0.G012590.1
HL.SW.v1.0.G021106.1
HL.SW.v1.0.G021105.1
HL.SW.v1.0.G009649.1
HL.SW.v1.0.G008530.1
HL.SW.v1.0.G000878.1
HL.SW.v1.0.G018728.1
HL.SW.v1.0.G010657.1
HL.SW.v1.0.G021104.1
HL.SW.v1.0.G034529.1
HL.SW.v1.0.G025856.1
HL.SW.v1.0.G035931.1
HL.SW.v1.0.G001728.1
HL.SW.v1.0.G041306.1
HL.SW.v1.0.G038119.1
HL.SW.v1.0.G019549.1
HL.SW.v1.0.G000619.1
HL.SW.v1.0.G005640.1
HL.SW.v1.0.G019335.1
HL.SW.v1.0.G000888.1
HL.SW.v1.0.G000208.1
HL.SW.v1.0.G031438.1
HL.SW.v1.0.G044172.1
HL.SW.v1.0.G014470.1
HL.SW.v1.0.G016378.1
HL.SW.v1.0.G008629.1
HL.SW.v1.0.G020466.1
HL.SW.v1.0.G011193.1
motif 1
motif 2
motif 3
motif 4
motif 5
motif 6
motif 7
motif 8
motif 9
motif 10
Motif 1
Motif 2
Motif 3
Motif 4
Motif 5
Motif 6
Motif 7
Motif 8
Motif 9
Motif 10

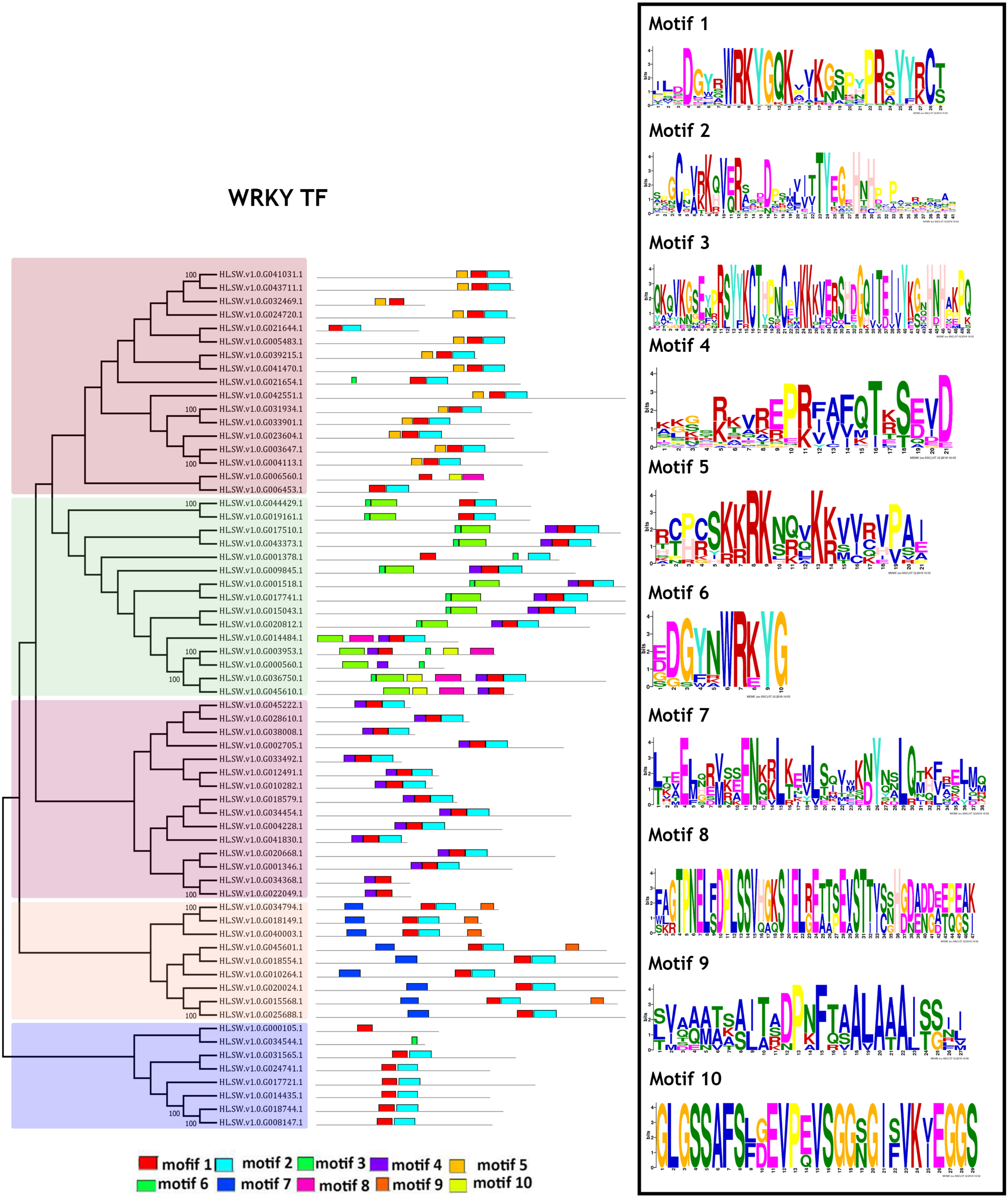
WRKY TF
HL.SW.v1.0.G041031.1
HL.SW.v1.0.G043711.1
HL.SW.v1.0.G032469.1
HL.SW.v1.0.G024720.1
HL.SW.v1.0.G021644.1
HL.SW.v1.0.G005483.1
HL.SW.v1.0.G039215.1
HL.SW.v1.0.G041470.1
HL.SW.v1.0.G021654.1
HL.SW.v1.0.G042551.1
HL.SW.v1.0.G031934.1
HL.SW.v1.0.G033901.1
HL.SW.v1.0.G023604.1
HL.SW.v1.0.G003647.1
HL.SW.v1.0.G004113.1
HL.SW.v1.0.G006560.1
HL.SW.v1.0.G006453.1
HL.SW.v1.0.G044429.1
HL.SW.v1.0.G019161.1
HL.SW.v1.0.G017510.1
HL.SW.v1.0.G043373.1
HL.SW.v1.0.G001378.1
HL.SW.v1.0.G009845.1
HL.SW.v1.0.G001518.1
HL.SW.v1.0.G017741.1
HL.SW.v1.0.G015043.1
HL.SW.v1.0.G020812.1
HL.SW.v1.0.G014484.1
HL.SW.v1.0.G003953.1
HL.SW.v1.0.G000560.1
HL.SW.v1.0.G036750.1
HL.SW.v1.0.G045610.1
HL.SW.v1.0.G045222.1
HL.SW.v1.0.G028610.1
HL.SW.v1.0.G038008.1
HL.SW.v1.0.G002705.1
HL.SW.v1.0.G033492.1
HL.SW.v1.0.G012491.1
HL.SW.v1.0.G010282.1
HL.SW.v1.0.G018579.1
HL.SW.v1.0.G034454.1
HL.SW.v1.0.G004228.1
HL.SW.v1.0.G041830.1
HL.SW.v1.0.G020668.1
HL.SW.v1.0.G001346.1
HL.SW.v1.0.G034368.1
HL.SW.v1.0.G022049.1
HL.SW.v1.0.G034794.1
HL.SW.v1.0.G018149.1
HL.SW.v1.0.G040003.1
HL.SW.v1.0.G045601.1
HL.SW.v1.0.G018554.1
HL.SW.v1.0.G010264.1
HL.SW.v1.0.G020024.1
HL.SW.v1.0.G015568.1
HL.SW.v1.0.G025688.1
HL.SW.v1.0.G000105.1
HL.SW.v1.0.G034544.1
HL.SW.v1.0.G031565.1
HL.SW.v1.0.G024741.1
HL.SW.v1.0.G017721.1
HL.SW.v1.0.G014435.1
HL.SW.v1.0.G018744.1
HL.SW.v1.0.G008147.1
motif 1
motif 2
motif 3
motif 4
motif 5
motif 6
motif 7
motif 8
motif 9
motif 10
Motif 1
Motif 2
Motif 3
Motif 4
Motif 5
Motif 6
Motif 7
Motif 8
Motif 9
Motif 10
