# Supplementary material for: Revisiting the Role of Transcription Factors in Coordinating the Defense Response Against Citrus Bark Cracking Viroid Infection in Commercial Hop (Humulus Lupulus L.)
Source: Viruses. 2019 May 5;11(5):419. doi: 10.3390/v11050419 (PMC6563305; doi:10.3390/v11050419)
Supplement: Supplementary file 1 [file viruses-11-00419-s001.zip › Supplementary Table 1_List of qPCR primers.doc]

| **Primer name** | **Gene name** | **Sequence (5’ – 3’)** | **GC** | **Tm** | **bp** | **Function** | **Reference** |
| --- | --- | --- | --- | --- | --- | --- | --- |
| ERF109-F | Ethylene response factor 109 | TGATAAGGGAGTTGAATCTGGG | 45 | 59.88 | 63 | JA-AUX crosstalk, retards PCD | Cai et al. 2014 |
| ERF109-R |  | GCAATCCATCATAGCCGTC | 52 | 59.30 |  |  |  |
| ERF_SHINE2-F | Ethylene-responsive transcription factor SHINE 2 | GCTGGAAAGAGTAGTAACGAC | 47 | 59 | 180 | Floral development | Shi et al. 2011 |
| ERF_SHINE2-R |  | TAAGGCAAGTGAGGGAAGGA | 50 | 60 |  |  |  |
| RT_bZIP61-F | Basic leucine zipper 61 | CTTGAGACGCCAATGCTG | 55 | 59.26 | 263 | Plant development | Shen et al. 2007 |
| RT_bZIP61-R |  | GTTGATGCTGTTATGATCGGAC | 45 | 59.95 |  |  |  |
| RT_RJ-F | Ribonuclease J | GGACCATTTGAAATAGAACCTCTG | 41 | 59.8 | 72 | mRNA cleavage | Sharwood et al. 2011 |
| RT_RJ-R |  | CACAGCGAAGAACTAACCC | 50 | 58.61 |  |  |  |
| RT_ASR3-F | ARABIDOPSIS SH4-  RELATED3 | GTCCTCATACAAGGTAAGCGA | 47 | 59.97 | 171 | Repression of immune gene | Hofmann 2015 |
| RT_ASR3-R |  | GTCTTTCCTCTGCAATTCCCA | 47 | 60.77 |  |  |  |
| RT_NAC78_F | NAC transcription factor 78 | ATGCGTTTGTGCTTTGTAGG | 45 | 59.5 | 148 | Biotic stress/flavanoid | Kan et al. 2017 |
| RT_NAC78_R |  | ATCACCAAGTAACACTTCACCA | 40 | 60 |  |  |  |
| RT_CUP_F | CUP-SHAPED COTYLEDON 3 | TGGTACTTCTTCAGCTTCAGG | 47 | 59.87 | 144 | Leaf deformation | Hasson et al. 2011 |
| RT_CUP_R |  | TCTTTCTCATGCCTACGATGTC | 45 | 59.13 |  |  |  |
| RT_WRKY68_F | Transcription factor WRKY 68 | TACTACGGCCACATCAACC | 52 | 59.57 | 198 | Resistance response | Shuo et al. 2016 |
| RT_WRKY68_R |  | TCTGGGTCTCATACTATTGGG | 47 | 59 |  |  |  |
| RT_WRKY40_F | Transcription factor WRKY 40 | AATGAACTAGCCTCCTTAACAC | 40 | 59 | 221 | Defense | Xu et al. 2007 |
| RT_WRKY40_R |  | GTATTCTTCTCCAGCCTTTCTC | 45 | 58.69 |  |  |  |
| RT_WRKY7_F | Transcription factor WRKY 7 | CCATCGCCGTGTATTTCTG | 52 | 59.19 | 107 | NA |  |
| RT_WRKY7_R |  | GACGAAGTGAGCTGAAACC | 52 | 59 |  |  |  |
| RT_MYC2_F | Transcription factor MYC2 | GACATCCAATCCCTCGTCAG | 55 | 60.37 | 186 | Boosts immune response | Pozo et al. 2008 |
| RT_MYC2_R |  | TCAACCAAACCAAAGAACCA | 40 | 59 |  |  |  |
